# Characterization of Detergent-Insoluble Proteins in ALS Indicates a Causal Link between Nitrative Stress and Aggregation in Pathogenesis

**DOI:** 10.1371/journal.pone.0008130

**Published:** 2009-12-02

**Authors:** Manuela Basso, Giuseppina Samengo, Giovanni Nardo, Tania Massignan, Giuseppina D'Alessandro, Silvia Tartari, Lavinia Cantoni, Marianna Marino, Cristina Cheroni, Silvia De Biasi, Maria Teresa Giordana, Michael J. Strong, Alvaro G. Estevez, Mario Salmona, Caterina Bendotti, Valentina Bonetto

**Affiliations:** 1 Dulbecco Telethon Institute, Milan, Italy; 2 Department of Molecular Biochemistry and Pharmacology, “Mario Negri” Institute for Pharmacological Research, Milan, Italy; 3 Department of Neuroscience, “Mario Negri” Institute for Pharmacological Research, Milan, Italy; 4 Department of Biomolecular Sciences and Biotechnology, University of Milan, Milan, Italy; 5 Department of Neuroscience, University of Turin, Turin, Italy; 6 Robarts Research Institute and Department of Clinical Neurological Sciences, University of Western Ontario, London, Ontario, Canada; 7 Burke Medical Research Institute, White Plains, New York, United States of America; Mayo Clinic, Jacksonville, United States of America

## Abstract

**Background:**

Amyotrophic lateral sclerosis (ALS) is a progressive and fatal motor neuron disease, and protein aggregation has been proposed as a possible pathogenetic mechanism. However, the aggregate protein constituents are poorly characterized so knowledge on the role of aggregation in pathogenesis is limited.

**Methodology/Principal Findings:**

We carried out a proteomic analysis of the protein composition of the insoluble fraction, as a model of protein aggregates, from familial ALS (fALS) mouse model at different disease stages. We identified several proteins enriched in the detergent-insoluble fraction already at a preclinical stage, including intermediate filaments, chaperones and mitochondrial proteins. Aconitase, HSC70 and cyclophilin A were also significantly enriched in the insoluble fraction of spinal cords of ALS patients. Moreover, we found that the majority of proteins in mice and HSP90 in patients were tyrosine-nitrated. We therefore investigated the role of nitrative stress in aggregate formation in fALS-like murine motor neuron-neuroblastoma (NSC-34) cell lines. By inhibiting nitric oxide synthesis the amount of insoluble proteins, particularly aconitase, HSC70, cyclophilin A and SOD1 can be substantially reduced.

**Conclusion/Significance:**

Analysis of the insoluble fractions from cellular/mouse models and human tissues revealed novel aggregation-prone proteins and suggests that nitrative stress contribute to protein aggregate formation in ALS.

## Introduction

Protein aggregation and deposits of abnormal proteins are hallmarks of several neurodegenerative diseases [1]. In familial forms the deposits frequently contain the mutant protein; in sporadic forms, post-translational modifications of proteins may be at the basis of the abnormal conformation. Aggregates are biochemically poorly characterized and what is known of the protein constituents comes essentially from immunohistochemistry studies. This is probably why their role in neurodegeneration remains poorly defined.

Amyotrophic lateral sclerosis (ALS) is a progressive and fatal motor neuron disease, and protein aggregation has been proposed as a possible pathogenetic mechanism [2]. Approximately 10% of ALS cases are familial; 20% of these are associated with mutations in the superoxide dismutase 1 (SOD1) gene. In SOD1-linked cases it is thought that the mutant protein acquires new toxic properties, such as the propensity to form aggregates [3], [4]. The aggregation hypothesis has received great support because mutant SOD1 mouse models of ALS develop protein inclusions in motor neurons and in some cases in astrocytes. In addition, insoluble SOD1 complexes can start to be detected prior to disease onset [5], [6]. Speculation has been offered on the mechanism of toxicity of SOD1-rich aggregates. For example, they may sequester other protein components essential for motor neuronal function, such as chaperones and anti-apoptotic molecules [7], inhibit the ubiquitin-proteasome system [8] and, by associating with motor proteins, impair axonal transport [9]. Insoluble mutant SOD1 was found associated with mitochondria and proposed as the basis of mitochondrial dysfunction [10].

In sporadic and familial ALS patients the most widely observed inclusions immunostain for ubiquitin, and other protein constituents are largely unknown [11]. Immunohistochemistry studies have detected proteins such as HSC70 [12], p38 MAP kinase [13] and TDP-43 [14] as constituents of the inclusions in ALS patients. In mutant SOD1 mice, protein inclusions are mainly immunoreactive for SOD1 and ubiquitin but also contain HSC70 and p38 MAPK [13]. We have shown that in the spinal cord of mice over-expressing hSOD1, carrying the G93A mutation (G93A SOD1 mice), there is progressive accumulation of mutant SOD1, its oligoubiquitinated forms and other unknown proteins in the Triton X-100-insoluble fraction (TIF) [5], [15]. We have now used proteomic approaches to characterize the protein composition of TIF, as a model of protein aggregates, in G93A SOD1 mice at different stages of disease. We identified several proteins enriched in TIF of ALS mice, most of them nitrated. Interestingly, we already detected increased protein nitration in the spinal cord soluble fraction of the G93A SOD1 mouse [16] and in the peripheral blood monuclear cells of ALS patients [17]. We therefore investigated the role of nitrative stress in aggregate formation in a cellular model of ALS and showed that by inhibiting nitric oxide synthesis it is possible to interfere with aggregation of proteins such as aconitase, HSC70, cyclophilin A (CypA) and SOD1.

## Results

In the spinal cord of G93A SOD1 mice we have observed progressive accumulation of Triton-insoluble proteins: mutant SOD1, its oligoubiquitinated forms and other unknown proteins [5], [15]. TIF from spinal cords of mutant mice are also enriched in polyubiquitinated proteins (Figure S1), and therefore have the fundamental biochemical features of protein inclusions in SOD1-linked ALS. For these reasons TIF was used as our experimental model of protein aggregates. In this study we characterized TIF of the spinal cord of G93A SOD1 mice at different disease stages.

### Proteomic Analysis of TIF from Spinal Cord of WT and G93A SOD1 Mice with Advanced Disease

**Figure 1:**
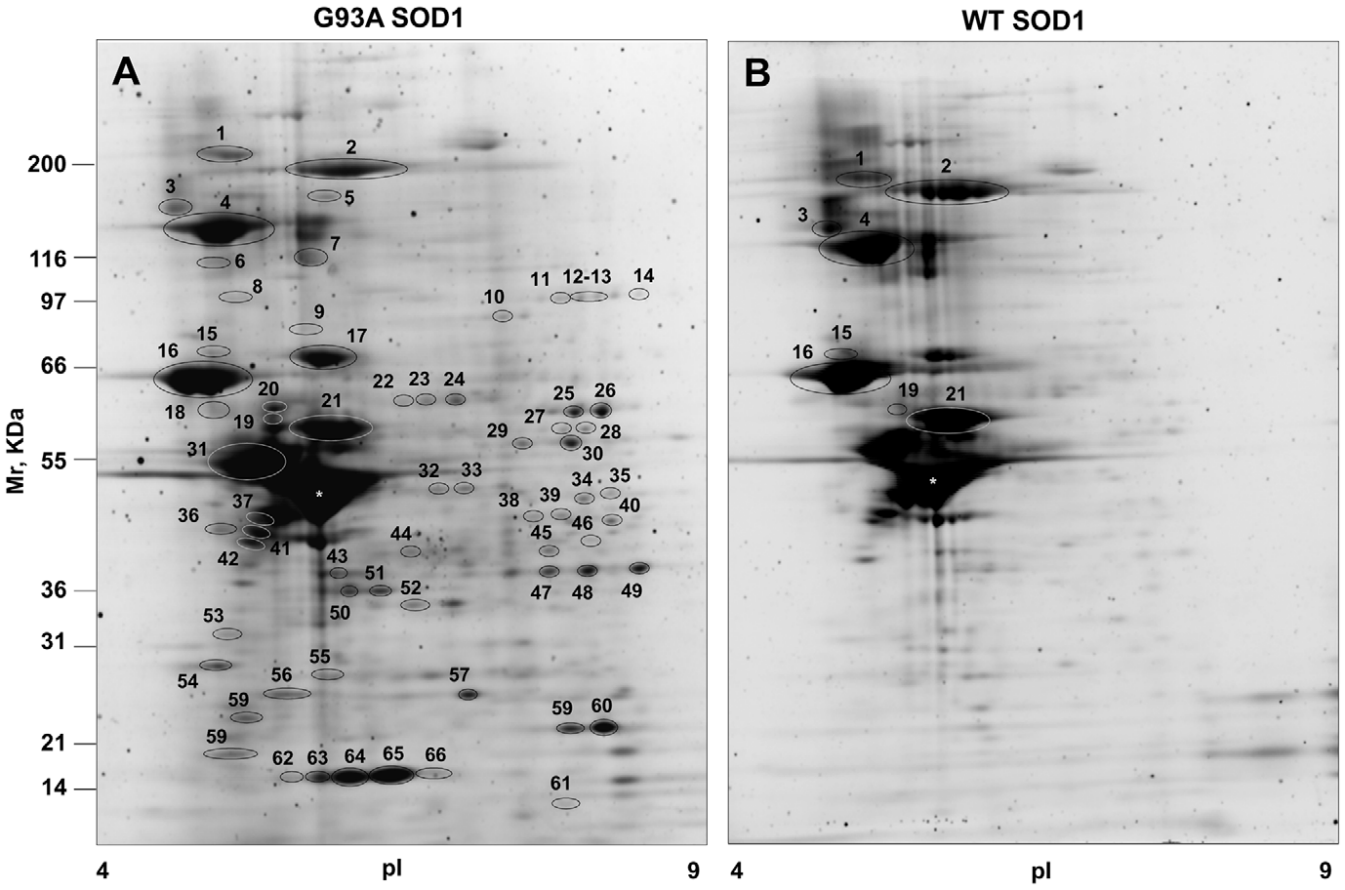
2DE proteomic analysis. Representative Sypro Ruby-stained 2DE maps of TIF of late-symptomatic G93A SOD1 mice (A) and age-matched WT SOD1 mice (B). In panel A the numbered spots correspond to proteins enriched or only present in TIF of G93A samples, and in panel B they indicate proteins enriched in TIF of WT samples. The same amount of protein was loaded in each gel (75 µg). The asterisk indicates the spot corresponding to GFAP, which is the most prominent, but equally abundant in the two conditions, and was therefore considered as background.

We started to analyze TIF from an advanced stage of disease, when protein aggregates are most abundant. TIF averaged 3.6±0.7 µg (n = 5) per mg of spinal cord tissue in G93A SOD1 mice at the end stage and 2.7±0.5 µg (n = 5) in age-matched wild-type (WT) SOD1 mice (p<0.05, as assessed by Student's t test). We analyzed the same amounts of TIF from spinal cord of G93A SOD1 mice and age-matched WT SOD1 mice by two-dimensional gel electrophoresis (2DE). Figure 1 shows 2-D average maps of G93A and WT samples. Gel images were analyzed and compared. The analysis detected changes in protein composition of TIF in the two conditions. There were 42 spots uniquely present in G93A samples (unmatched) and 94 spots with different volumes in G93A in comparison with WT samples; 62 were more present in G93A samples and 32 more present in WT samples. We defined the proteins similarly present in both samples as intrinsically poorly soluble in non-ionic detergents (the background), and the ones enriched or only present in G93A samples as protein aggregate constituents. After comparison of gel patterns 136 differentially present spots were excised from the gels and processed for protein identification.

### Identification of Differentially Present Proteins by MALDI-TOF

**Figure 2:**
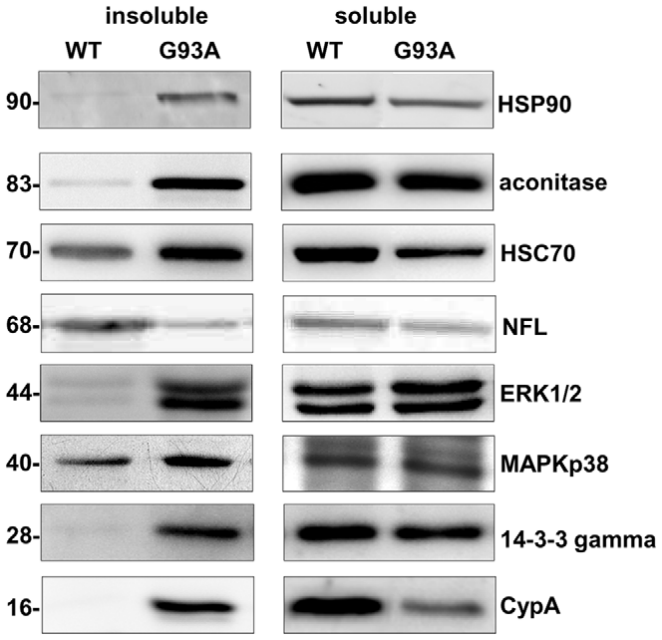
Immunoblot validation of TIF in mouse samples. Representative immunoblot of TIF and corresponding soluble fraction from spinal cord of late-symptomatic G93A SOD1 mice and age-matched WT SOD1 mice. Same amounts of TIF and soluble proteins (30 µg) were loaded in each immunoblot and probed with the specific antibodies. Immunoreactivity was normalized to the actual amount of protein loaded as detected after Coomassie blue staining.

Peptide mass fingerprinting spectra were recorded on a MALDI-TOF mass spectrometer and proteins identified by a database search using the MASCOT program. The proteins enriched or only present in TIF from G93A samples are reported in Table 1 and Table S1. They belong to different functional categories: cytoskeletal proteins, metabolic enzymes, mitochondrial proteins, chaperones, proteins involved in signalling and mutant SOD1. MAPKp38, previously found by immunohistochemistry in the inclusions in spinal motor neurons of these mice [13], was enriched in TIF of G93A SOD1 mice by Western blotting (WB) with the specific antibody (Fig. 2). The most abundant protein spot in the 2D gels (labelled with the asterisk in Fig. 1) was GFAP, which was not differentially present. Fragments (spot 37,41,42,43,55,56) and a high-Mw isoform (spot 5) of GFAP were instead specifically enriched in G93A samples. Some proteins were more present in TIF from WT mice and were therefore selected for protein identification (Table S2). We could identify intermediate filament proteins that are known to be enriched in TIF and more present in WT samples since equal amounts of total proteins for WT and G93A samples were loaded in the 2D gels. Clearly, the lower level of neurofilaments in G93A samples is correlated with the consistent motor neuron loss in G93A SOD1 mice with advanced disease.

**Table 1:** Proteins enriched in TIF of spinal cord from G93A SOD1 mice at 26 weeks of age compared to age-matched WT SOD1 mice.

Spot	Protein name	WT^a^	G93A^b^	FC^c^
	**Cytoskelton**			
5	**Glial fibrillary acidic protein (GFAP)** *	**-**	1.7±0.3	
20	**Vimentin** *	**-**	3.9±1.1	
56	GFAP#	0.8±0.1	2.9±0.6	3.6
58	Neurofilament triplet M protein# (NFM)	0.9±0.4	2.3±1.0	2.5
42	GFAP#	0.8±0.2	1.8±0.1	2.2
36	Vimentin#	1.5±0.1	3.0±1.0	2.0
31	Vimentin	47.6±7.1	78.6±14	1.7
37	GFAP#	5.2±0.2	8.3±0.9	1.6
55	GFAP#	1.7±0.3	2.8±0.7	1.6
41	GFAP#	3.2±0.4	4.8±0.6	1.5
43	GFAP#	4.1±0.6	6.2±0.6	1.5
	**Metabolism**			
32	**Alpha enolase**	**-**	1.1±0.3	
33	**Alpha enolase**	**-**	0.8±0.3	
39	**Glutamine synthetase**	**-**	0.7±0.2	
40	**Aspartate aminotransferase**	**-**	0.6±0.1	
46	**Fructose-bisphosphate aldolase C (aldolase)**	**-**	0.4±0.1	
49	**Glyceraldehyde-3-phosphate dehydrogenase (GAPDH)**	**-**	2.3±0.5	
51	**L-lactate dehydrogenase B chain (LDH)**	**-**	2.9±0.6	
26	Pyruvate kinase isozyme M2	0.2±0.1	2.7±0.7	13.5
25	Pyruvate kinase isozyme M2	0.3±0.0	2.4±0.4	8.0
52	Cytosolic malate dehydrogenase	0.3±0.2	1.4±0.2	4.7
48	GAPDH	0.6±0.1	2.8±0.6	4.6
47	GAPDH	0.5±0.3	1.9±0.3	3.8
38	Glutamine synthetase	0.3±0.0	0.8±0.3	2.7
	**Mitochondria**			
9	**NADH-ubiquinone oxidoreductase**	**-**	1.6±0.6	
10	**Glycerol-3-phosphate dehydrogenase**	**-**	0.3±0.1	
12–13	**Aconitase**	**-**	0.4±0.1	
14	**Aconitase**	**-**	0.3±0.0	
35	**Creatine kinase**	**-**	06±0.2	
44	**Isocitrate dehydrogenase [NAD]**	**-**	0.6±0.0	
34	Creatine kinase	0.2±0.1	1.0±0.2	5.0
30	ATP synthase alpha chain (ATPase)	0.6±0.0	2.3±0.6	3.8
11	Aconitase	0.2±0.1	0.6±0.1	3.0
50	Pyruvate dehydrogenase E1	1.4±0.3	3.7±0.3	2.6
27	Glutamate dehydrogenase 1	0.3±0.0	0.7±0.1	2.3
28	Glutamate dehydrogenase 1	0.3±0.1	0.7±0.1	2.3
29	ATPase	0.5±0.1	1.1±0.2	2.2
	**Chaperones**			
8	**Heat shock protein 90-alpha (HSP90)**	**-**	1.2±0.1	
59	**Alpha crystallin B chain**	**-**	1.7±0.6	
60	**Alpha crystallin B chain**	**-**	5.8±1.6	
61	**Peptidyl-prolyl cis-trans isomerase A (CypA)**	**-**	0.4±0.1	
57	Heat-shock protein beta-1 (HSP27)	0.7±0.1	3.2±0.9	4.7
17	Heat shock cognate 70 kDa protein (HSC70)	10.0±2.2	16.4±4	1.6
	**Signaling**			
53	**Annexin A5**	**-**	1.1±0.2	
54	14-3-3 protein gamma	1.1±0.2	3.9±1.1	3.4
45	ERK2	0.3±0.04	0.9±0.2	2.7
	**Endoplasmic reticulum**			
6	**Endoplasmin**	**-**	2.6±0.5	
7	**Transitional endoplasmic reticulum ATPase**	**-**	5.9±1.3	
18	**Protein disulfide-isomerase (PDI)**	**-**	2.4±0.4	
	**Others**			
62	**SOD1**	**-**	0.9±0.1	
63	**SOD1**	**-**	3.3±0.3	
64	**SOD1**	**-**	8.4±1.1	
65	**SOD1**	**-**	14.5±2.2	
66	**SOD1**	**-**	1.6±0.2	
23	Dihydropyrimidinase-related protein 2	0.7±1.3	1.4±0.4	2.0
24	Dihydropyrimidinase-related protein 2	0.8±0.0	1.6±0.3	2.0
22	Dihydropyrimidinase-related protein 2	0.8±0.2	1.5±0.4	1.9

The proteins are categorized by their known function, in bold are the ones only found in G93A samples, the others are sorted by their fold change from highest to lowest.

a WT, normalized spot volumes of the WT sample, mean of three replicates±SD.

b G93A, normalized spot volumes of the G93A samples, mean of three replicates±SD.

c FC, fold change of spot volume as ratio of the spot volumes (G93A/WT); the value is missing for proteins only found in G93A samples.

-spot not detected in WT samples.

* Mr higher than expected, unknown protein modification.

# Mr lower than expected, possible protein fragment.

### Validation Analysis in Mouse and Human Spinal Cord Samples

Some of the proteins enriched in TIF of G93A samples were selected for validation by WB: HSP90, aconitase, HSC70, ERK2, 14-3-3 gamma, and CypA. Fig. 2 shows representative WB of the same amounts of TIF from spinal cord of WT and G93A SOD1 mice probed with the specific antibodies. In all cases the enrichment of the proteins analyzed by 2DE was confirmed by WB (Fig. 2, Table S3). The levels of these proteins were also measured in the soluble fraction. HSP90, aconitase, ERK1/2, 14-3-3 gamma were similarly present in the soluble fraction of spinal cord of WT and G93A SOD1 mice, while HSC70 and CypA, abundantly expressed in neurons [18], [19], were substantially lower in G93A SOD1 mice, probably because of motor neuron loss.

**Figure 3:**
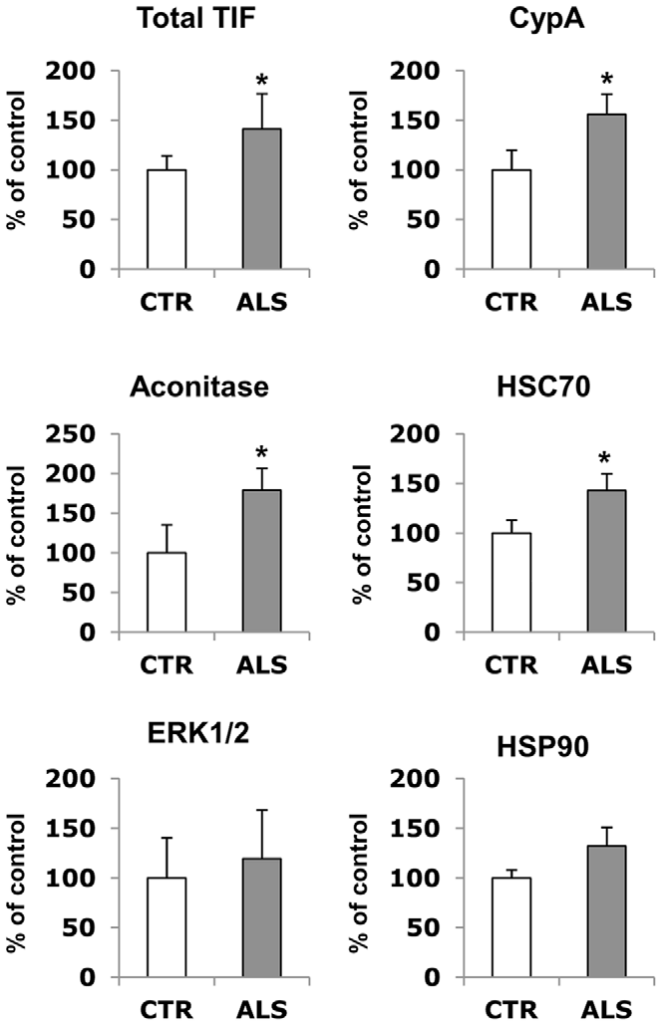
Immunoblot validation of TIF in human samples. Dot blot analysis of TIF in spinal cord tissues of sporadic ALS patients (n = 7), black bars, and controls (CTR) (n = 3), white bars. Total TIF is the ratio of the amount of TIF to the total proteins extracted. Proteins were quantified by the BCA protein assay. The same amount of TIF (3 µg) was loaded on the membrane and probed with the specific antibodies. Histograms represent the immunoreactivity normalized to the actual amount of protein loaded, detected after Red Ponceau staining. Values are percentages of controls and are the mean±SD. *, significantly different from controls as assessed by unpaired t test with Welsh's correction (*p*<0.05).

TIF was also extracted from spinal cord tissues of sporadic ALS patients and controls. Significantly more TIF was obtained from patients than controls averaging 2.3±0.3 µg (n = 7) in comparison with 1.7±0.5 µg (n = 3) per mg of tissue analyzed (p<0.05, as assessed by unpaired t test with Welsh's correction). The levels of HSP90, aconitase, HSC70, ERK1/2 and CypA were measured by dot blot analysis. Interestingly, CypA, aconitase, and HSC70 were significantly enriched in TIF of patients (Fig. 3). The level of the same proteins in the soluble fraction did not change (data not shown).

### DIGE Analysis of TIF from Spinal Cord of G93A SOD1 Mice at Different Stages of Disease

We then measured the levels of aggregated proteins at earlier disease stages, pre-symptomatic and early symptomatic. TIF from spinal cord of G93A SOD1 mice at the three different stages was analyzed by DIGE and compared with TIF from spinal cord of WT SOD1 mice (Figure S2 and Table S4). Of the 66 protein spots analyzed, 35 were more present in the G93A samples than in WT already at 12 weeks of age, while 19 accumulated only at the end-stage. For example, the neurofilament proteins L (NFL) and M (NFM) accumulated in TIF of G93A SOD1 mice at 12 weeks of age, while at end-stage disease the level of the insoluble proteins fell, parallel with the motor neuron loss. Mitochondrial proteins such as NADH-ubiquinone oxidoreductase and aconitase accumulated at all ages as much as chaperone proteins, HSP90 and HSC70. Insoluble 14-3-3 protein gamma was not recovered in TIF of WT mice but was present at all ages in G93A SOD1 mice. Proteins involved in glycolytic pathways, fructose-bisphosphate aldolase C (aldolase C) and glyceraldehyde-3-phosphate dehydrogenase (GAPDH), greatly accumulated, only or especially at end-stage disease, as well as ERK2.

### Immunohistochemistry of Aconitase

**Figure 4:**
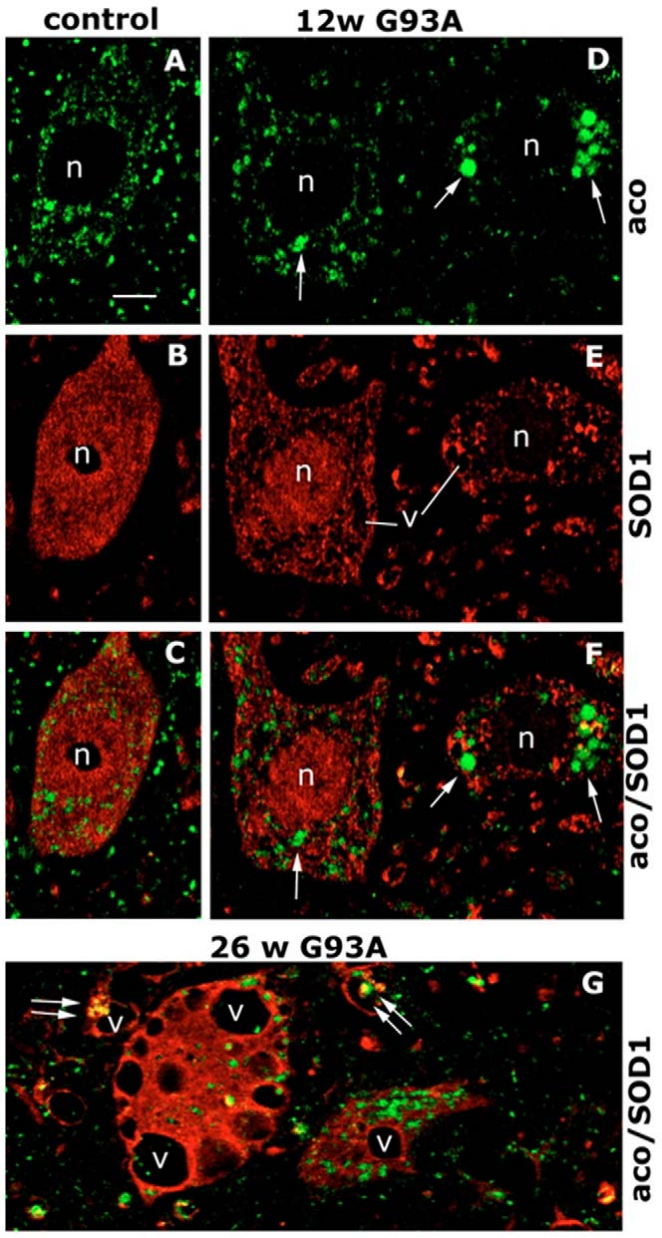
Immunohistochemistry of aconitase. Immunolabelling for aconitase (A, C, D, F, G, green) and human SOD1 (B, C, E–G, red) in ventral horn lumbar spinal cord sections from a 26-week-old control WT SOD1 mouse (A–C) and G93A SOD1 mice at 12 (D–F) and 26 (G) weeks. C, F, G are merged images. In controls the human SOD1-expressing motor neurons show fine punctate aconitase labelling (A–C), whereas in presymptomatic G93A SOD1 mice the motor neurons expressing mutant human SOD1 show large aggregates of aconitase (D, arrows) only partially overlapping SOD1-positive aggregates (F). In end-stage G93A SOD1 mice (G) aggregates of aconitase and SOD1 are present in large vacuolated (v) motor neurons and also in the surrounding neuropil (double arrows); n, nuclei; v, vacuoles. Bar = 40 µm.

**Figure 5:**
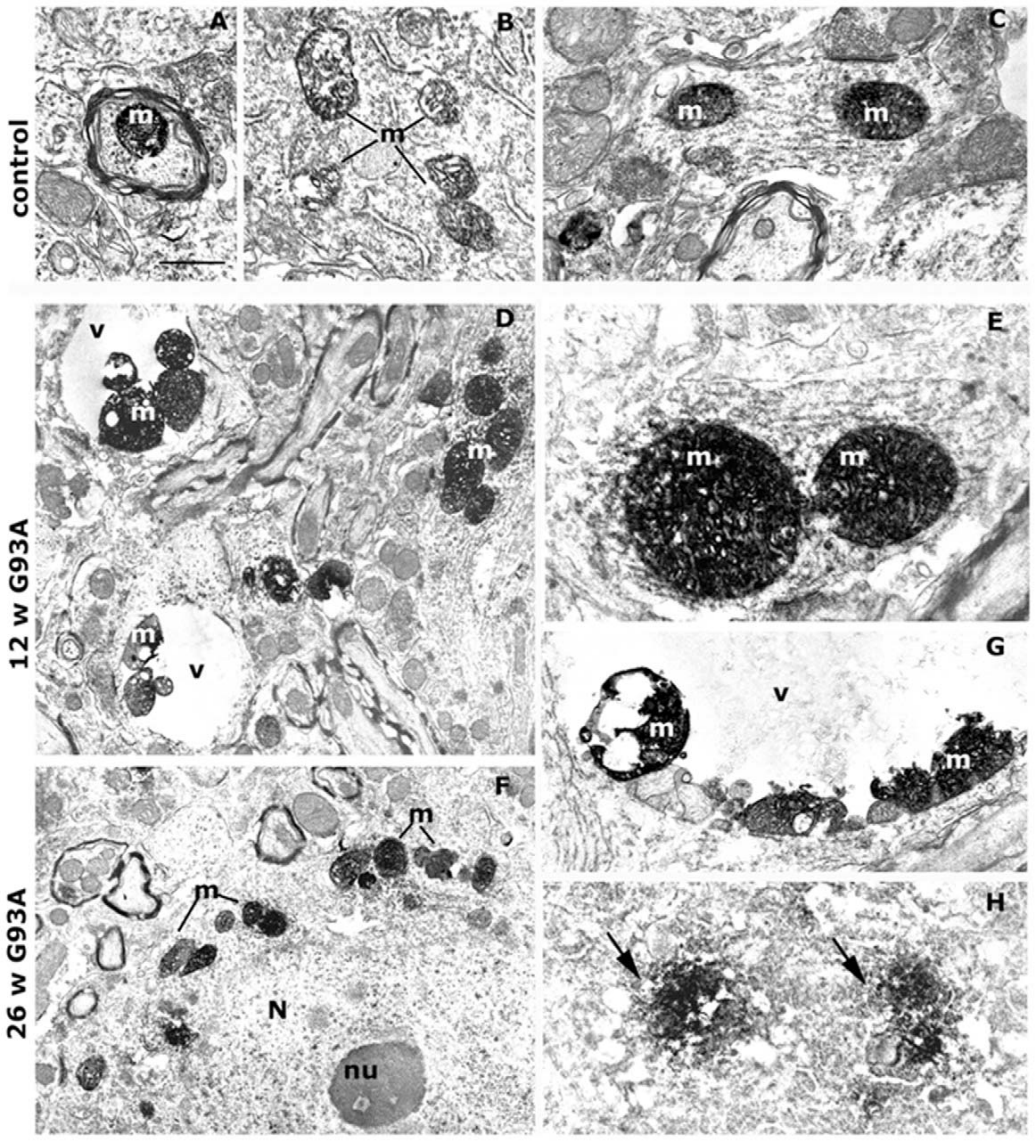
Ultrastructural localization of aconitase. Immunolabelling in the ventral horn of non-trasgenic control mice at 26 weeks of age (A–C) and of G93A SOD1 mice at pre-symptomatic (12 weeks, D–E) and end-stage (26 weeks, F–G) of disease. In controls labelling is in mitochondria (m) located in axons (A), cell bodies (B) and dendrites (C). In G93A SOD1 mice intensely labelled mitochondria (m) are clustered together in dendrites (D) and cell bodies (F), swollen (E) and apposed at the membrane of vacuoles (v) (D, G). Occasional clumps of aconitase-positive material (arrows) are found in end-stage motor neurons (H). N, Nucleus; nu, nucleolus. Bars: A–C, E = 1 µm; D, F = 2,5 µm; G = 1,2 µm; H = 1,4 µm.

To verify the localization of proteins identified in the proteomic screening, we did immunostaining analysis on spinal cord sections of G93A SOD1 mice at pre-symptomatic and end stages of disease. We selected aconitase that had no known prior association with inclusions in ALS. Double labelling with anti-aconitase and anti-cytochrome oxidase in the lumbar spinal cord of control samples (Figure S3) showed that the punctate labelling of aconitase and that of the mitochondrial marker cytochrome oxidase largely overlapped in neuronal cell bodies and profiles scattered in the neuropil. Aconitase immunoreactivity in human SOD1-labelled motor neurons of WT SOD1 control mice (Fig. 4A–C) was similar to that in non-transgenic mice (data not shown) and uniformly distributed in small puncta around the nucleus. In contrast, in human SOD1-labelled motor neurons of G93A SOD1 mice aconitase immunoreactivity was found in large puncta already at the pre-symptomatic stage of disease (Fig. 4D–F) and at the end stage it occasionally co-localized with human SOD1 also in neuropilar aggregates (Fig. 4G). Electron microscopy confirmed that the anti-aconitase antiserum used selectively labelled mitochondria in spinal cord samples of control and transgenic mice (Fig. 5). In the ventral horn of non-transgenic mice labelled mitochondria were present in myelinated axons, cell bodies and dendrites, close to unlabelled mitochondria (Fig. 5A, B, C). In G93A SOD1 mice at both pre-symptomatic (Fig. 5D, E) and end-stage (Fig. 5F–G) ages, an intense aconitase staining was found in numerous mitochondria located in dendrites (Fig. 5D, E, G) and cell bodies (Fig. 5F). Several labelled mitochondria appeared swollen (Fig. 5E) and were frequently aggregated in clusters or apposed at the inner membrane of vacuoles (Fig. 5D, G). Only in end-stage G93A SOD1 mice the anti-aconitase antiserum also labelled clumps of amorphous material scattered in the cytoplasm of large neuronal cell bodies identifiable as motor neurons (Fig. 5H).

### The Majority of the Insoluble Proteins Are Tyrosine Nitrated

**Figure 6:**
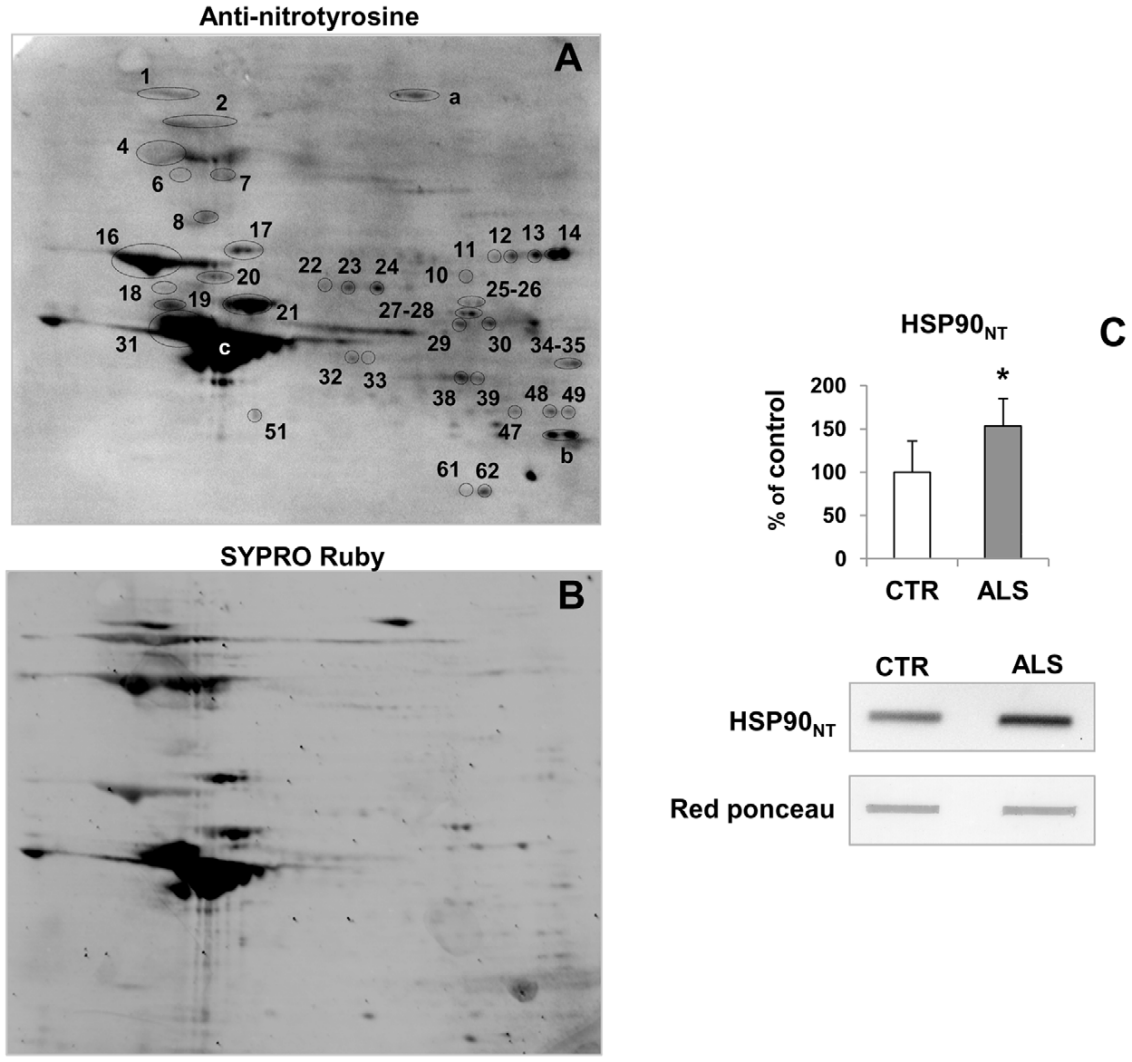
Analysis of nitrated proteins in TIF of 17-week-old G93A SOD1 mice. 150 µg of TIF was loaded into the 2D gel and transferred onto a PDVF membrane. The blot was probed with anti-nitrotyrosine polyclonal antibody (A), after total protein SYPRO Ruby blot staining (B). Nitrated protein signals of the 2D WB were matched and localized in a twin 2D gel and proteins were identified by peptide mass fingerprinting. Spot numbers in (A) correspond to proteins in Table 2. a, b and c are spots corresponding respectively to laminin subunit beta-2, VDAC, GFAP, that were not specifically increased in G93A TIF in the proteomic screening. (C) Dot blot analysis of nitrated HSP90 in TIF of spinal cord tissues of controls (CTR) (n = 3), white bars, and sporadic ALS patients (n = 7), grey bars. The same amount of TIF (3 µg) was loaded on the membrane and probed with the specific antibody. Histograms represent the immunoreactivity normalized to the actual amount of protein loaded, as detected after Red Ponceau staining. Values are percentages of controls and are the mean±SD. *, significantly different from controls as assessed by unpaired t test with Welsh's correction (*p*<0.05). Representative dot blots for a control and an ALS patient are reported.

We have previously shown a high level of protein nitration in the soluble fraction of the spinal cord of G93A SOD1 mice already at a pre-symptomatic stage of the disease, increasing as the disease progresses [16]. We took into consideration that protein nitration may be involved in the aggregation by altering the protein structure and stability. We analyzed protein nitration in spinal cord TIF at early symptomatic and end-stage disease. Figure 6 shows a representative 2D WB of TIF from early symptomatic G93A SOD1 mice probed with anti-nitrotyrosine polyclonal antibody. Similar results were obtained with the monoclonal anti-nitrotyrosine antibody. Surprisingly, the majority of the protein spots in TIF, 39 out of 69 (Table 2), were nitrated and gave a very intense signal (Fig. 6A), especially the mitochondrial protein aconitase (spots 11,12,13,14), HSC70 (spot 17) and the intermediate filament proteins, NFL (spot 16), alpha-internexin (spot 21), vimentin (spot 31) and GFAP (spot c); NFM (spot 1,4) and NFH (spot 2), although abundant (Fig. 6B), were only mildly nitrated (Fig. 6A). Nitrated proteins in TIF from WT SOD1 samples were hardly detected (Figure S4). To check whether there is a parallel with the human disease, the level of nitrated HSP90 (spot 8 in the mouse experiment), for which a specific antibody is available [20], was measured in the TIF of sporadic ALS patients and controls by dot blot analysis. Interestingly, the TIF of ALS patients showed enrichment of the nitrated protein (Fig. 6C). The level of nitrated HSP90 in the soluble fraction was not changed (data not shown).

**Table 2:** Nitrated proteins in TIF of spinal cord of G93A SOD1 mice.

Spot	Protein name
	**Cytoskeleton**
1	NFM*
2	NFH
4	NFM
16	NFL
19–20	Vimentin*
21	Alpha-internexin
31	Vimentin
	**Metabolism**
25–26	Pyruvate kinase isozyme M2
32–33	Alpha enolase
38–39	Glutamine synthetase
47–48–49	GAPDH
51	LDH
	**Mitochondria**
10	Glycerol-3-phosphate dehydrogenase
11–12–13–14	Aconitase
27–28	Glutamate dehydrogenase 1
29–30	ATPase
34–35	Creatine kinase
	**Chaperones**
8	HSP90
17	HSC70
59–60	Alpha crystallin B chain
	**Endoplasmic reticulum**
6	Endoplasmin
7	Transitional endoplasmic reticulum ATPase
18	PDI
	**Others**
22–23–24	Dihydropyrimidinase-related protein 2
a	Laminin subunit beta-2
b	VDAC1
c	GFAP

* Mr higher than expected, unknown protein modification; a, b, c, proteins not specifically enriched in TIF of G93A mice.

### L-NAME Reduces the Level of Detergent-Insoluble Proteins in a Cellular Model of fALS

**Figure 7:**
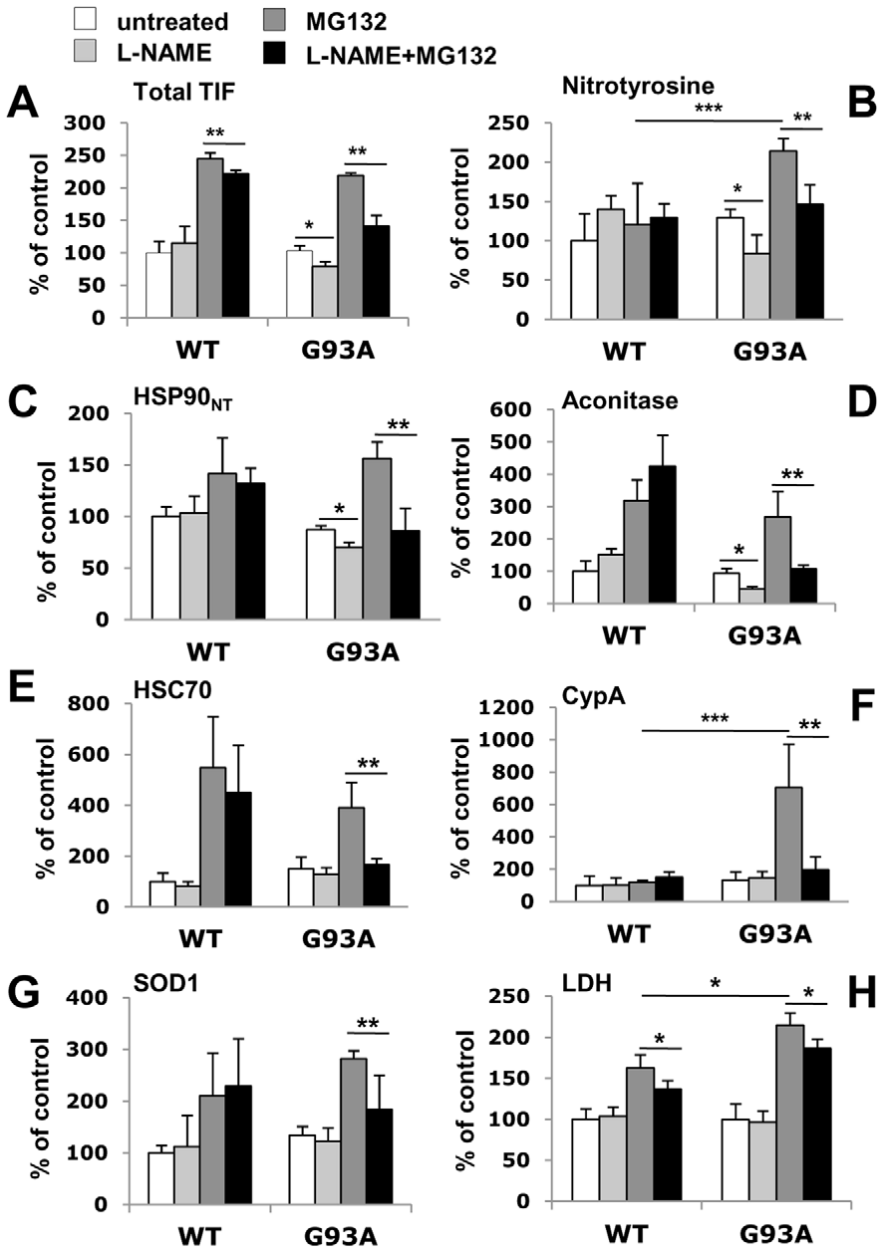
Analysis of TIF of NSC-34 cells expressing WT and G93A hSOD1, treated or not with MG132, L-NAME or both. (A) Total TIF is the ratio of the amount of TIF to the total proteins extracted. Proteins were quantified in each condition by the BCA protein assay. Values are percentages of the untreated WT control and are the mean±SEM (n = 3). (B–G) The level of nitrotyrosine, nitrated HSP90 (HSP90_NT_), aconitase, HSC70, CypA and SOD1 were measured by dot blot analysis. The same amount of cellular TIF (3 µg) was loaded on the membrane and probed with the specific antibodies. Histograms represent the immunoreactivity normalized to the actual amount of protein loaded, as detected after Red Ponceau staining, multiplied by the total TIF isolated for each condition. Values are percentages of the untreated WT control and are the mean±SEM (n = 3). *, significantly different from untreated G93A controls (*p*<0.05); **, significantly different from MG132-treated G93A samples (*p*<0.05); ***, significantly different from MG132-treated WT samples (*p*<0.05), as assessed by one-way ANOVA followed by Newman-Keuls multiple comparison test. (H) Analysis of cell death by quantification of extracellular LDH activity. Histograms represent mean±SD of four replicates. One-way ANOVA was followed by Newman-Keuls multiple comparison test. *, *p*<0.05.

To investigate whether protein nitration has a causative role in aggregate formation or is just a consequence of the longer exposure of protein inclusions to oxidative stress, we used the NSC-34 cell line expressing G93A hSOD1, or WT hSOD1 as control. These cells did not produce evident aggregates under basal conditions, however they were induced to accumulate insoluble proteins by treatment with a proteasome inhibitor (MG132), similarly to previous findings [21]. Under these conditions double the amount of TIF was isolated from WT and G93A SOD1 expressing cells compared to untreated cells (Fig. 7A). The cellular TIF from mutant SOD1 cells had the biochemical features of the one isolated from the spinal cord of mutant SOD1 mice: high levels of mutant SOD1 (Fig. 7G), nitrated proteins (Fig. 7B) and ubiquitinated proteins (data not shown). This enabled us to examine the *ab initio* aggregate formation of some of the proteins found in the TIF of the mice, also present in the cellular TIF. We measured the effect of a non-selective nitric oxide synthase (NOS) inhibitor, L-NAME, on the insolubility of aconitase and HSC70, nitrated in the mice, CypA and SOD1, susceptible to other types of oxidative modifications [22]–[24], and nitrated HSP90 (Fig. 7C–G). Fig. 7A–B shows that L-NAME reduced the total MG132-induced TIF in NSC-34 cells expressing G93A SOD1 by 56% and this reduction paralleled the reduction of nitrated proteins (52%). Specifically, L-NAME reduced the amount of nitrated HSP90 by 81%, aconitase by 72%, HSC70 by 86%, CypA by 91% and SOD1 by 61% in TIF of G93A SOD1 NSC-34 cells (Fig. 7C–G). L-NAME also had an effect in TIF isolated from G93A SOD1 cells under basal conditions, and although small it was significant for nitrated HSP90 and aconitase (Fig. 7C,D). The reduction of MG132-induced TIF in WT SOD1 cells was smaller, 18%, and was never significant for the single proteins analyzed. It is noteworthy that MG132 alone did not raise the level of nitrated proteins in TIF from WT SOD1 cells (Fig. 7B). These data suggest that the increase in nitrated proteins in G93A SOD1 cells has to be attributed to the increased oxidative/nitrative stress caused, directly or indirectly, by mutant SOD1 [25]–[28]. The L-NAME treatment was therefore effective only in G93A SOD1 cells possibly because only there oxidative/nitrative stress played a role in the formation and consolidation of the aggregates. We measured cell death by quantifying extracellular LDH activity in cells lines expressing WT and G93A SOD1, treated with MG132, L-NAME or both (Fig. 7H). As expected, MG132 was toxic on both cell lines, but was significantly more toxic in G93A SOD1 expressing cells. Interestingly, L-NAME in combination with MG132 partially rescued cells from MG132-induced toxicity, reducing cell death by respectively 16% and 13% in WT and G93A cells.

## Discussion

We previously reported that mutant SOD1 and its oligoubiquitinated forms are abundantly recovered in TIF of the spinal cord from G93A SOD1 mice [5]. However, from that study we deduced that there were several unknown proteins in addition to mutant SOD1. The present proteomic analysis enabled us to identify 66 protein spots exclusively present or more abundant in TIF of ALS mice than controls. To our knowledge, this is the first successful large-scale analysis of detergent-insoluble proteins in an ALS mouse model. This was possible because of the use of an optimized 2DE-based proteomic approach to isolate and analyze TIF. A previous attempt, based on liquid chromatography-electrospray ionization mass spectrometry, found primarily SOD1 and only traces of other abundant proteins [29].

It is known that mutant SOD1 forms aggregates in different cellular compartments such as mitochondria [10], endoplasmic reticulum [30] and perykaria [31]. We found insoluble proteins from these subcellular compartments, and showed that many of these proteins start to aggregate already at a presymptomatic stage of the disease as much as mutant SOD1 [5].

Intermediate filaments such as neurofilaments, vimentin and GFAP were the most abundant proteins recovered in TIF of WT and G93A SOD1 mice. However, high-Mw isoforms of NFM, NFL, vimentin and GFAP were only found in TIF of ALS mice. These may be ubiquitinated forms, but because of their low abundance we were not able to identify the modification by mass spectrometry. Immunocolocalization of ubiquitin and neurofilaments has already been observed in neuronal hyaline inclusions in G93A SOD1 mice and ALS patients [32], [33]. Fragments of intermediate filaments already accumulated at a pre-symptomatic stage of the disease. GFAP and NFL fragments have been observed in spinal cords of ALS patients [34], [35] and this may indicate increased activation of specific proteases or oxidation-induced protein fragmentation [36].

Several enzymes important in energy metabolism were also present. Their aggregation may explain the defective mitochondrial respiratory chain activities and ATP production in the mutant mice [37]. While glycolytic enzymes are highly recovered mainly at symptomatic stages of disease, insoluble mitochondrial enzymes already accumulate at a pre-symptomatic stage. This agrees with the observations of early alterations of mitochondria [38] and the presence of SOD1-rich aggregates in mitochondria of ALS mice [10]. Among the mitochondrial enzymes, mitochondrial aconitase, which is altered in aging and neurodegenerative diseases it is of special interest [39], [40]. The enzyme is highly sensitive to oxidative inactivation and modifications [41]. We have reported that aconitase is susceptible to tyrosine nitration, as detected in the soluble fraction of the spinal cord of pre-symptomatic G93A SOD1 mice [16]. In this study, we found it was abundantly recovered, highly nitrated, in TIF. Accumulation is substantial already before the onset of disease, as confirmed by immunostaining analysis on spinal cord sections. In some cases it co-localized with SOD1, however it is likely that it can aggregate also independently from G93A SOD1. Accumulation of the insoluble protein was also detected in spinal cord tissues of sporadic ALS patients. This confirms a mitochondrial alteration in the animal model and in patients. It also candidates aconitase as a sensitive biomarker of the human disease.

One of the functional categories highly present in our analysis is the chaperone. Chaperones are potent controllers of protein aggregation, promoting protein folding and refolding, and cooperating to degrade irreversibly damaged proteins. They were greatly enriched in TIF of G93A SOD1 mice early in the disease and, except for HSC70, absent or scant in WT SOD1 mice. A specific interaction between chaperones and mutant SOD1, but not WT SOD1, is also indicated in other works [42], [43]. Chaperone activity has been reported to be reduced in spinal cord of G93A and G85R SOD1 mice before the disease onset [44]. One possibility is that chaperones are sequestered by misfolded mutant SOD1, so are less available for cytoprotective functions. This notion is borne out by the fact that increasing expression of HSP70 by gene transfer protected cultured motor neurons from mutant SOD1 toxicity [45], although overexpressing only HSP70 was not effective *in vivo* [46]. As suggested by our analysis, which found several chaperones damaged, upregulating a panel of such proteins is likely to be a more successful pharmacological strategy.

Proteins involved in signalling were also enriched early in the disease. 14-3-3 protein gamma is a protein adaptor that recognize the phosphoserine-containing motif of several target proteins and regulates signal transduction pathways. 14-3-3 proteins have been found in Lewy body-like hyaline inclusions in ALS patients [47]. These proteins may recognize the phosphorylated serine residues of neurofilaments and promote their abnormal accumulation, or remain entrapped in the inclusions. A similar situation may arise with ERK. ERK1/2 are MAP kinases, which are activated by various mechanisms and have more than 100 different substrates, including NFM, NFH and alpha crystalline [48]. It is possible that ERK1/2 are aberrantly activated and sequestered with the substrates in the aggregates. Finally, TDP-43 was not found among the aggregated proteins in the G93A SOD1 mice, as already reported in another study [49].

What is peculiar is that most of the proteins found in TIF are intrinsically soluble and stable with no apparent reason to be co-purified with insoluble mutant SOD1. The high affinity of chaperones for mutant misfolded SOD1 only partially explains the molecular determinants of aggregation. We have shown that the level of proteins carrying an oxidative modification, tyrosine nitration, possibly induced by mutant SOD1 [25], [26], are increased in the spinal cord soluble fraction of G93A SOD1 mice already at a pre-symptomatic stage of disease [16]. Interestingly, some of these nitrated proteins were also recovered in TIF, including HSC70, alpha enolase and ATPase. Nitrated NFL has been shown to inhibit the assembly of unmodified neurofilament subunits and therefore may be at the basis of neurofilament aggregate formation [50]. Nitrated alpha synuclein and tau have been found in brain of patients with Parkinson's and Alzheimer's diseases [51], [52]. However, *in vitro*, at least for alpha synuclein, the impact of nitration on aggregation is controversial [53], [54].

Since no comprehensive study of the nitration pattern of insoluble proteins has ever been done, it was not possible to consider protein nitration as a potential general mechanism of protein aggregation. By using a proteomic approach we demonstrated that the majority of the proteins enriched in TIF of the ALS mouse was nitrated. In human tissues at least one nitrated protein, HSP90, was detected enriched in TIF of sporadic ALS patients. Thus nitration might have some role in aggregate formation in ALS. Nevertheless, from such experiments *ex vivo* we could not establish whether nitration was a consequence of the longer life-time of proteins entrapped in the inclusions. Using a NSC-34 cell model of ALS we demonstrated that inhibiting nitrative stress by treatment with a non-selective NOS inhibitor, L-NAME, substantially reduced the amount of MG132-induced insoluble proteins. This confirms a previous study showing that treatments with a proteasome inhibitor of cell lines expressing mutant SOD1 decreased aggregation of certain proteins [21], strengthening the hypothesis that nitration plays a role in aggregation. More specifically, we detected reduced levels of insoluble nitrated HSP90, aconitase and HSC70, nitrated in the mouse samples, CypA and SOD1, susceptible to cysteine thiol modifications [24], [55], [56]. Inhibition of NO synthesis leads to a decrease in peroxynitrite formation, which in turn may reduce tyrosine nitration but also various cysteine oxidations, including disulfides and nitrosothiols. We therefore propose that L-NAME interferes more generally with oxidative modification-induced protein aggregation in the presence of mutant SOD1. Under this condition the reported decrease in the level of endogenous antioxidants might play a role [28], [57]. However, in this cell paradigm we could not really evaluate the effect of reduced protein aggregation on cell viability. L-NAME only partially rescued cells from MG132 treatment, which is highly toxic at the concentration used to induce aggregate formation. It has been reported that *in vivo* treatments with NOS inhibitors were protective in animal models of motor neuron degeneration, but in other studies they were ineffective [58]–[60]. Although the role of NOS and the use of NOS inhibitors for therapeutic purposes is debated [59], [61], [62], our data provide additional indications of the importance of aiming pharmacological approaches at pathways that modulate nitrative stress which, if regulated as early as possible, may influence downstream aggregation pathways too.

In conclusion, a striking difference between WT and G93A SOD1 mice is in TIF and consists in the portion of the proteome that, damaged or altered in pathological conditions, loses its structural determinants and accumulates as insoluble material as the disease progresses. Some components of this insoluble fraction are also found in sporadic ALS patients suggesting that they could be novel markers of the human sporadic forms. Finally, characterization of tyrosine nitrated insoluble proteins showed that nitrative stress, induced by SOD1 mutation or other unknown instigation factor(s) in the case of the sporadic forms, may contribute to protein aggregate formation in ALS.

## Materials and Methods

### Transgenic Mice

Transgenic G93A SOD1 mice originally obtained from Jackson Laboratories and expressing about 20 copies of mutant human (h)SOD1 with a Gly93Ala substitution (B6SJL-TgNSOD-1-SOD1G93A-1Gur), or WT hSOD1 were bred and maintained on a C57BL/6 genetic background at Harlan Italy S.R.L., Bresso (MI), Italy. Transgenic mice were identified by PCR. The mice were housed at 21±1°C with 55±10% relative humidity and 12 h light. Food (standard pellets) and water were supplied *ad libitum*. Female G93A SOD1 mice were sacrified at pre-symptomatic (12 weeks of age), early symptomatic (17 weeks of age) and end-stages (26 weeks) of the disease. Female non transgenic littermates and WT SOD1 mice at 26 weeks of age were used as controls. Procedures involving animals and their care were conducted in conformity with the institutional guidelines that are in compliance with national (D.L. No. 116, G.U. Suppl. 40, Feb. 18, 1992, Circolare No. 8, G.U., 14 luglio 1994) and international laws and policies (EEC Council Directive 86/609, OJ L 358,1, Dec.12, 1987; NIH Guide for the Care and Use of Laboratory Animals, U.S. National Research Council, 1996).

### Human Samples

Frozen spinal cord from controls and ALS patients were partly from the Netherlands Brain Bank (NBB), Netherlands Institute for Neuroscience, Amsterdam, and partly provided by Michael Strong, Robarts Research Institute, London, Ontario. Post-mortem delay of the control subjects was <12 h and of ALS patients was <12 h (n = 3), <24 h (n = 4). No abnormalities were detectable at autopsy in the spinal cord tissues of the three controls who died due to cardiac arrest, cancer and pneumonia. All ALS cases were negative for mutations in TDP-43 and SOD1. Table S5 reports the clinical and neuropathological characteristics of the ALS cases. All material has been collected and used in compliance with the ethical and legal declaration of the Netherlands Brain Bank and Robarts Research Institute after a written informed consent from donor or legal representative.

### Extraction of Detergent-Insoluble Protein

Tissues were processed as previously described [5]. Briefly, they were homogenized in ice-cold homogenisation buffer, pH 7.6, containing 15 mM Tris-HCl, 1 mM DTT, 0.25 M sucrose, 1 mM MgCl_2_, 2.5 mM EDTA, 1 mM EGTA, 0.25 M sodium orthovanadate, 2 mM sodium pyrophosphate, 5 µM MG132 proteasome inhibitor (Sigma), 1 tablet of Complete™/10 mL of buffer, Mini Protease Inhibitor Mixture (Roche Applied Science). The samples were centrifuged at 10000×g at 4°C for 15 minutes, obtaining a supernatant (S1) and a pellet. The pellet was suspended in ice-cold homogenisation buffer with 2% of Triton X-100 and 150 mM KCl, sonicated three times for 10 sec and shaken for 1 hour at 4°C. Samples were then centrifuged twice at 10000×g at 4°C for 10 minutes to obtain Triton X-100-resistant pellets (TIF) and a supernatant (S2). The soluble fraction is considered the pool of S1 and S2 fractions. Proteins were quantified by the Bradford assay. To isolate TIF from human spinal cords, tissues were cut with a cryostat microtome and the sections were collected in a tube containing 10 volumes (w/v) of homogenisation buffer and processed as described for the mice tissues. To isolate TIF from cells the protocol was slightly modified. Briefly, cells were directly lysed in 0.2% of Triton X-100 and 150 mM KCl, sonicated and shaken for 1 hour at 4°C. Samples were then centrifuged at 100,000×g for 1 hour. The pellets were boiled in 50 mM Tris HCl pH 6.8 and SDS 2% and analyzed. Proteins were quantified by the BCA protein assay (Pierce).

### 2DE

Samples were dissolved in 7 M urea, 2 M thiourea, 4% (w/v) CHAPS, 0.5% (v/v) IPG buffer (GE Healthcare) and 12 µg/mL DeStreak™ Reagent (GE Healthcare). Samples were pools of TIF from five mice for each genotype. Aliquots of 75 µg were loaded in each 2D gel by in-gel rehydration (1 h at 0 V, 270 Vhr at 30 V) on pH 3-10 non-linear 7-cm IPG strips (GE Healthcare). IEF was done on an IPGphor (GE Healthcare) according to the following schedule: 200 Vhr at 200 V, 925 Vhr of a linear gradient up to 3500 V, 10500 Vhr at 3500 V, 14375 Vhr of a linear gradient up to 8000 V, 48000 Vhr at 8000 V. Strips were then re-equilibrated in NuPAGE LDS Sample Buffer (Invitrogen) and second dimension was run on precast, 4–12% polyacrylamide gradient gel, NuPAGE® Bis-Tris (Invitrogen). Gels were stained with SYPRO® Ruby protein gel stain (Invitrogen).

### 2D Image Analysis and Quantification

Changes in protein spot volumes were calculated comparing gels from pools of five samples, from 26-week-old WT and G93A SOD1 mice, run in triplicate. Gel images were captured by the laser scanner Molecular Imager® FX (Bio-Rad) and 2D image analysis was done with Progenesis PG240 v2006 software (Nonlinear Dynamics). The analysis protocol for the gel images included: spot detection, warping, background subtraction, averaged gel creation, matching and reference gel modification. Detection, warping, and matching of the protein spots were done using the “combined warp and match” algorithm, which uses a nonparametric pattern recognition clustering technique to align different gel images. The “Total spot volume normalization” algorithm was used to calculate each protein spot volume as the sum of the intensities of the pixels within the spot's boundary, minus the background level within that same boundary, normalized to the total spot volumes in the gel. Observed pI and Mr were calculated by the software based on protein spots of known characteristics.

### Protein Identification

Protein spots were located and excised with an EXQuest™ spot cutter (Bio-Rad). Spots were processed and gel-digested with trypsin, as previously described [16]. Tryptic digests were concentrated and desalted using ZipTip pipette tips with C18 resin and 0.2 µl bed volume (Millipore). Peptide mass fingerprinting was done on a ReflexIII™ MALDI-TOF mass spectrometer (Bruker Daltonics) equipped with a SCOUT 384 multiprobe inlet and a 337-nm nitrogen laser using α-cyano-4-hydroxycinnamic acid as matrix, prepared as previously described [63]. All mass spectra were obtained in positive reflector mode with a delayed extraction of 200 ns. The reflector voltage was set to 23 kV and the detector voltage to 1.7 kV. All the other parameters were set for an optimized mass resolution. To avoid detector saturation low-mass material (500 Da) was deflected. The mass spectra were internally calibrated with trypsin autolysis fragments. The mass spectra were obtained by averaging 150–350 individual laser shots and then automatically processed by the FlexAnalysis software, version 2.0 using the following parameters: the Savitzky Golay smoothing algorithm and the SNAP peak detection algorithm. Database searches (Swiss-Prot, release 57.3, June 2009) were done using the Mascot software package available on the net (http://www.matrixscience.com), allowing up to one missed trypsin cleavage, carbamidomethylation of Cys and oxidation of Met, as variable modifications, and a mass tolerance of ±0.1 Da over all *Mus musculus* protein sequences deposited. A protein was regarded as identified if the following criteria were fulfilled: the probability-based MOWSE [64] score was above the 5% significance threshold for the database and the spots excised from at least two different gels gave the same identification.

### DIGE Analysis

The four experimental groups were: G93A SOD1 mice at 12, 17 and 26 weeks of age and WT SOD1 mice at 26 weeks of age. Equal amounts of TIF from spinal cords of five mice from each group were pooled. Samples were labelled according to the manufacturer's instructions (GE Healthcare) with minor modifications. Briefly, 25 µg of each pool was labelled with 200 pmol of Cy3 or Cy5 dye for 30 min in ice in the dark. To exclude preferential labelling of the dyes, each sample was also reverse labelled. As an internal standard, aliquots of each pool were mixed and labelled with Cy2 dye. Four 2D gel were run as described in the 2DE section. Each gel contained two experimental groups, one Cy3-labelled, the other Cy5-labelled plus the Cy2-labelled internal standard. Gel images were captured by the laser scanner Molecular Imager FX (Bio-Rad). Image analysis was done with Progenesis PG240 v2006 software (Nonlinear Dynamics). The spots analyzed were those that were differentially expressed in the end-stage analysis. For each spot the normalized volume was standardized against the intra-gel standard, dividing the value for each spot normal volume by the corresponding internal standard spot normal volume within each gel. The values for each spot in each group were expressed as mean the of the values from the Cy3- and Cy5-labelled analyses.

### WB

Proteins were transferred onto PVDF membranes (Millipore). For the reaction with the primary antibodies, membranes were incubated for 1 h at room temperature with a blocking buffer (5% milk in Tris-buffered saline containing 0.1% Tween 20) and probed overnight at 4°C. Primary antibodies were: rabbit polyclonal anti-mitochondrial aconitase (1∶2500), kindly provided by Dr. L.I. Szweda [65], rabbit polyclonal anti-14-3-3 protein gamma (1∶2000), kindly provided by Dr. F. Tagliavini, rabbit polyclonal anti-heat shock protein 90 (HSP90) (1∶1000) from Stressgen, mouse monoclonal anti-heat shock cognate 70 kDa protein (HSC70) (1∶1000) from Santa Cruz Biotechnology, mouse monoclonal anti-neurofilament triplet L protein (NFL) (1∶1000) and rabbit polyclonal anti-CypA (1∶2500) from Upstate, rabbit polyclonal anti-ERK1/2 (1∶1000), rabbit polyclonal anti-MAP kinase p38 (MAPKp38) (1∶1000) from Cell Signaling, and rabbit polyclonal anti-ubiquitin (1∶800) from DAKO. The blots were probed with goat anti-rabbit or anti-mouse peroxidase-conjugated secondary antibodies (Santa Cruz Biotechnology) and developed by the ECL Plus protein detection system (GE Healthcare) or Immobilon Western Chemiluminescent HRP Substrate (Millipore) on the ChemiDoc XRS system (Bio-Rad). Densitometry was done with Progenesis PG240 v2006 software (Nonlinear Dynamics). Immunoreactivity was normalised to the actual amount of proteins loaded on the membrane as detected after Coomassie Blue staining.

### Analysis of TIF from Human Samples

Total TIF is considered the ratio of the amount of isolated TIF to the total proteins extracted. Proteins were quantified by the BCA protein assay (Pierce). An aliquot of TIF (3 µg) from the post-mortem samples was loaded on nitrocellulose membrane, Trans-Blot Transfer Medium (Bio-Rad), by vacuum deposition on the Bio-Dot SF blotting apparatus (Bio-Rad). Membranes were probed with the specific primary antibodies and then with goat anti-rabbit or anti-mouse peroxidase-conjugated secondary antibodies (Santa Cruz Biotechnology). Blots were developed by Immobilon Western Chemiluminescent HRP Substrate (Millipore) on the ChemiDoc XRS system (Bio-Rad). Densitometry was done with Progenesis PG240 v2006 software (Nonlinear Dynamics). Immunoreactivity was normalised to the actual amount of proteins loaded on the membrane as detected after Red Ponceau staining (Fluka).

### Immunohistochemistry

Female mice (at least four for each group) were anesthetized with Equitensin (1% phenobarbital/4% (vol/vol) chloral hydrate, 6 µL/g, ip) and transcardially perfused with 20 mL of sodium phosphate buffer (PBS) followed by 50 mL 4% paraformaldehyde solution in PBS. Spinal cords were rapidly removed and post-fixed as previously described [66]. Immunolabelling was done on lumbar spinal cord sections (30-µm thick floating cryosections or 40-µm thick vibratome sections). Endogenous peroxidases were inactivated by 1% hydrogen peroxide in PBS (135 mM NaCl, 2.6 mM KCl, 10 mM Na_2_HPO_4_, 1.76 mM KH_2_PO_4_, pH 7.4). The sections were incubated with 5% normal goat serum (NGS) in PBST (PBS + 0.3% Triton X-100) 1 h at RT, then probed overnight at 4°C in 5% NGS, PBST with a rabbit polyclonal anti-aconitase antibody (1∶500, kindly provided by Dr. L.I. Szweda). Subsequently the sections were washed in PBS and incubated 1 h at RT in 5% NGS, PBST with a secondary biotinylated anti-rabbit antibody, diluted 1∶200, from Vector. The secondary antibody was revealed with a TSA amplification kit, Cy5 (Perkin Elmer) as previously described [66]. For SOD1 labelling the mouse monoclonal anti-human SOD1, MO62-3 (clone 1G2, 1∶3000, MBL, Japan) was used and for mitochondrial labelling the mouse monoclonal anti cytochrome oxidase subunit I antibody (clone 1D6, 1∶200, Molecular Probes) was used. Fluorescence-labelled sections were mounted with Fluorsave (Calbiochem) and analyzed under an Olympus Fluoview or a TCS NT Leica laser scanning confocal microscope. Selected vibratome sections, permeabilised with ethanol instead of Triton, were processed for the ultrastructural detection of aconitase using a standard immunoperoxidase procedure using the Vectastain ABC kit (Vector) and diaminobenzidine tetrahydrochloride (DAB) as a chromogen. After visualization of reaction product with DAB, sections were osmicated, dehydrated and flat-embedded in epoxy resin. Selected areas of the embedded sections were then cut with a razor blade and glued to blank blocks of resin for further sectioning with an ultramicrotome. Thin sections collected on copper grids were counterstained with lead citrate and observed and photographed with a Zeiss 902 electron microscope.

### Detection and Identification of Nitrated Proteins

TIF from WT and G93A samples were loaded on 7-cm IPG strips (pH 3–10, non-linear) and separated by IEF as described in the 2DE section, in duplicate. One 2D gel was stained with SYPRO® Ruby protein gel stain (Invitrogen) and the other was transferred onto PVDF membrane (Millipore) and probed overnight at 4°C with anti-nitrotyrosine rabbit polyclonal antibody, provided by A.G. Estevez, or the anti-nitrotyrosine mouse monoclonal antibody (clone HM.11; HyCult Biotechnology). Results were visualized with the Qdot® 800 goat anti-rabbit or anti-mouse IgG conjugate antibody (Invitrogen), capturing the images with the laser scanner Molecular Imager FX (Bio-Rad). Nitrated protein signals of the 2D WB were localized in the 2D gel by the specific warping algorithm of the Progenesis software and processed for identification by peptide mass fingerprinting. No false immunopositive spots were detected as tested by treating membranes with sodium dithionite, as described previously [16]. Dot blot analysis of TIF of human tissues was done with anti-nitrated HSP90 monoclonal antibody, prepared and characterized as described [20].

### NSC-34 Cell Lines

WT or G93A SOD1 expressing NSC-34 cells were obtained by stably transfecting a NSC-34 derived line expressing the tetracycline-controlled transactivator protein tTA with pBI-EGFP containing WT or G93A hSOD1 cDNA cloned downstream to the tetracycline-responsive bidirectional promoter [67] and express similar amount of human and murine SOD1 (Figure S5). Cell lines were kept in culture in DMEM (high glucose) supplemented with tet-screened FBS (5%, Clontech), 1 mM glutamine, 1 mM pyruvate, antibiotics (100 IU/mL penicillin and 100 µg/mL streptomycin), G418 sulphate (0.5 mg/mL) (Invitrogen) and hygromycin (0.2 mg/mL) (Invitrogen). For this study cells were grown without doxycycline in the medium, allowing full expression of transfected WT or G93A SOD1.

### Cell Treatments

A 10-mM stock solution of MG132 (Calbiochem) was prepared in dimethylsulfoxide and a 30-mM stock solution of L-NAME (Sigma) in PBS. Cells were seeded at a density of 6850 cells/cm^2^ in T25 flasks, and grown under standard conditions for six days, then treated with MG132, L-NAME or both (respectively 5 µM and 300 µM final concentration). After 24 h, the cells were detached with 1xPBS and washed once with 1xPBS, then recovered by centrifugation at 250×g for 10 minutes. The cell pellets were stored at −80°C until analysis of protein aggregates.

### Analysis of Cellular TIF

Three cell pellets from different flasks for each genotype and condition were independently processed to obtain the TIF. A sample of 3 µg TIF for each condition was loaded on nitrocellulose membrane and analyzed on the Bio-Dot SF blotting apparatus (Bio-Rad) with the antibodies for the specific proteins, as described for the human samples. Immunoreactivity values were multiplied by the total TIF from each cell pellet. Total TIF is considered the ratio of the amount of isolated TIF to the total proteins extracted. Proteins were quantified by the BCA protein assay (Pierce).

### Cytotoxicity Assay

Cell death was analyzed by quantifying extracellular lactate dehydrogenase (LDH) activity with the cytotoxicity detection kit of LDH (Roche Applied Science), as described [68]. For each sample, the ratio of extracellular to intracellular LDH activities was obtained. Results were expressed as percentages of the untreated control cells of each cell line.

## Supporting Information

Figure S1 Representative anti-ubiquitin immunoblot of TIF from late-symptomatic G93A SOD1 and age-matched WT SOD1 mice. The same amount of TIF (30 µg) was loaded in each immunoblot.
(1.41 MB TIF)

Figure S2 Representative Cy-dye 2DE maps of TIF from spinal cord of G93A SOD1 mice at 12, 17 and 26 weeks of age in comparison with WT SOD1 mice. The same amount of protein was loaded (75 µg) in each gel and contained a Cy3-labelled sample (25 µg), a Cy5-labelled sample (25 µg) and the Cy2-labelled internal standard (25 µg). Gel images were captured by the laser scanner Molecular Imager FX (Bio-Rad). Image analysis was done with Progenesis PG240 v2006 software (Nonlinear Dynamics). The spots considered in the analysis were the ones found differentially expressed in the end-stage analysis (Fig. 1 and Table 1).
(7.70 MB TIF)

Figure S3 Lumbar ventral horn of a non-transgenic mouse labeled for anti-aconitase (A, green) and anti-cytochrome oxidase (B, red). Both markers form a fine punctate labeling pattern in the soma of motor neurons (n, nucleus) and in the neuropil. C. Merged images show colocalization (yellow). Bar = 40 µm.
(0.47 MB TIF)

Figure S4 Analysis of nitrated proteins in TIF of 26-week-old WT SOD1 mice: 150 µg of TIF was loaded into the 2D gel and transferred onto a PDVF membrane. The blot was probed with anti-nitrotyrosine polyclonal antibody (A), after total protein SYPRO Ruby blot staining (B). Spot numbers in (A) correspond to proteins in Table 2.
(5.69 MB TIF)

Figure S5 Anti-SOD1 Western blot of total protein extracts from NSC-34 cells expressing WT or G93A SOD1. The same amount of proteins (30 µg) was loaded in immunoblot.
(0.90 MB TIF)

Table S1 MALDI TOF MS identification of proteins differentially present in TIF from spinal cord of WT and G93A SOD1 mice.
(0.12 MB DOC)

Table S2 Proteins enriched in TIF of spinal cord from WT SOD1 mice.
(0.04 MB DOC)

Table S3 WB of selected proteins. The proteins were measured in TIF (aggregate) and in the soluble (soluble) fraction of spinal cord protein extracts from WT and G93A SOD1 mice at 26 weeks of age.
(0.11 MB DOC)

Table S4 DIGE analysis of TIF from spinal cord of WT and G93A SOD1 mice at different disease stages.
(0.10 MB DOC)

Table S5 Clinical and neuropathological characteristics of ALS cases.
(0.03 MB DOC)
