# Supplementary figures and images for: Characterization of Detergent-Insoluble Proteins in ALS Indicates a Causal Link between Nitrative Stress and Aggregation in Pathogenesis
Source: PLoS One. 2009 Dec 2;4(12):e8130. doi: 10.1371/journal.pone.0008130 (PMC2780298; doi:10.1371/journal.pone.0008130)

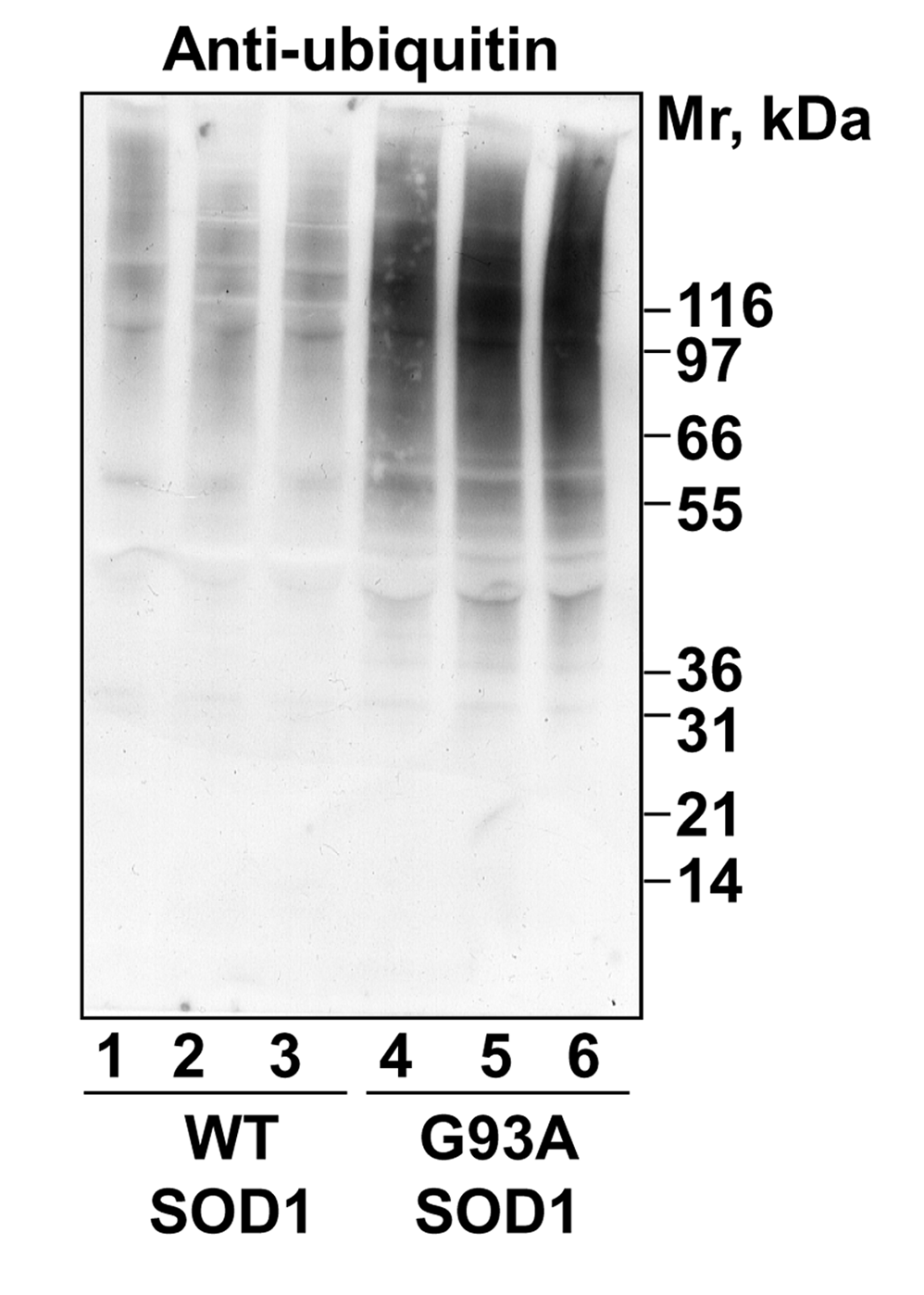

Supplement: Figure S1 — Representative anti-ubiquitin immunoblot of TIF from late-symptomatic G93A SOD1 and age-matched WT SOD1 mice. The same amount of TIF (30 µg) was loaded in each immunoblot. (1.41 MB TIF) [file pone.0008130.s001.tif]

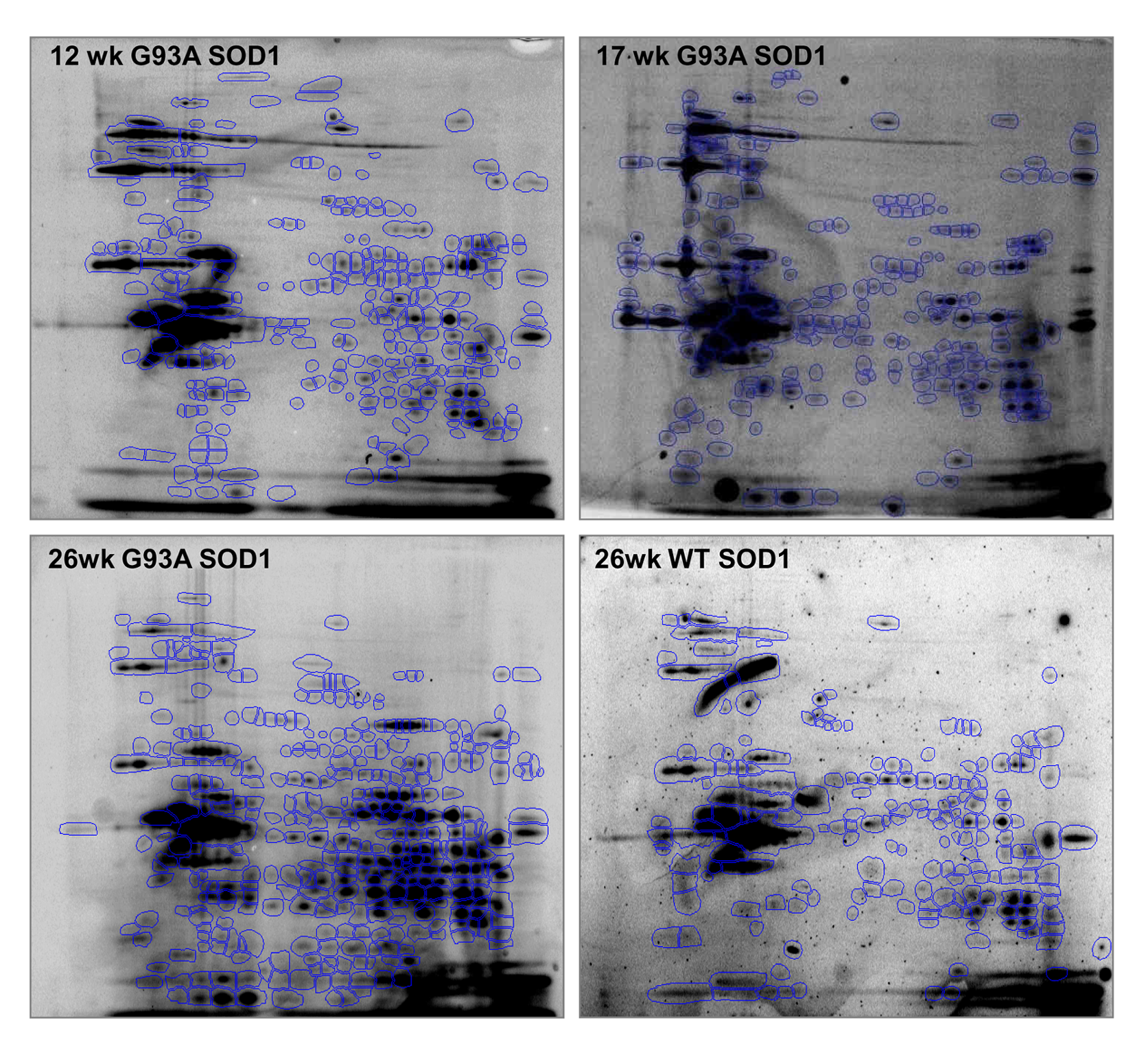

Supplement: Figure S2 — Representative Cy-dye 2DE maps of TIF from spinal cord of G93A SOD1 mice at 12, 17 and 26 weeks of age in comparison with WT SOD1 mice. The same amount of protein was loaded (75 µg) in each gel and contained a Cy3-labelled sample (25 µg), a Cy5-labelled sample (25 µg) and the Cy2-labelled internal standard (25 µg). Gel images were captured by the laser scanner Molecular Imager FX (Bio-Rad). Image analysis was done with Progenesis PG240 v2006 software (Nonlinear Dynamics). The spots considered in the analysis were the ones found differentially expressed in the end-stage analysis (Fig. 1 and Table 1). (7.70 MB TIF) [file pone.0008130.s002.tif]

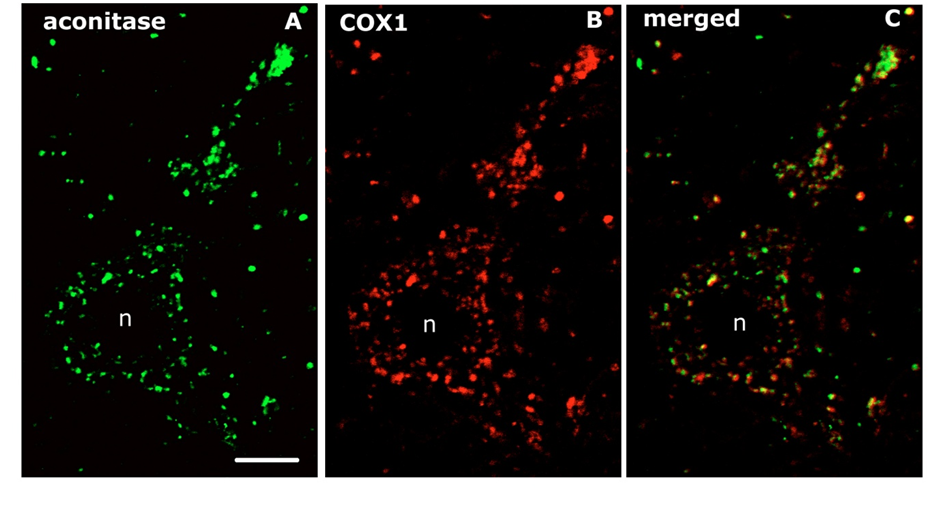

Supplement: Figure S3 — Lumbar ventral horn of a non-transgenic mouse labeled for anti-aconitase (A, green) and anti-cytochrome oxidase (B, red). Both markers form a fine punctate labeling pattern in the soma of motor neurons (n, nucleus) and in the neuropil. C. Merged images show colocalization (yellow). Bar = 40 µm. (0.47 MB TIF) [file pone.0008130.s003.tif]

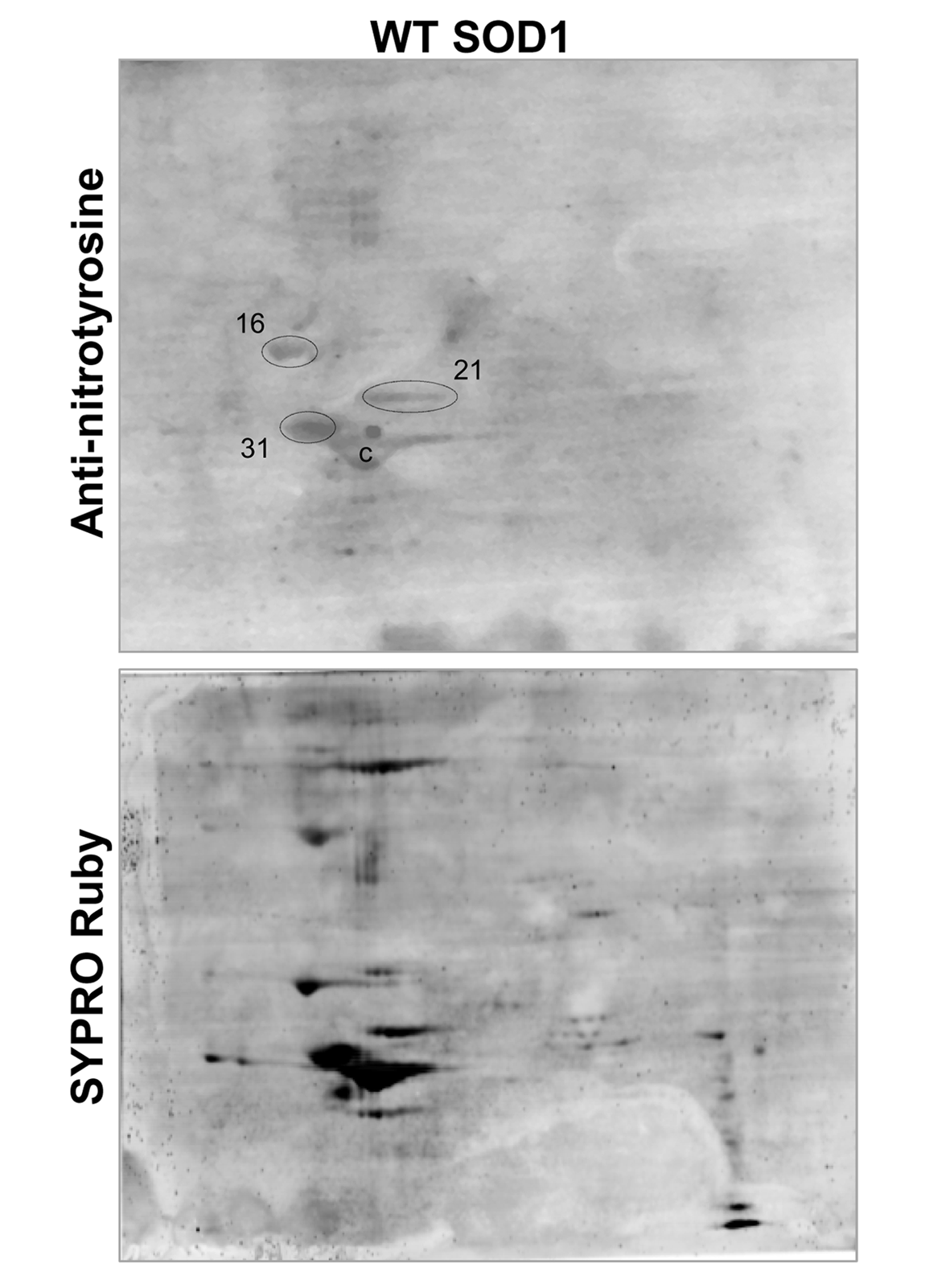

Supplement: Figure S4 — Analysis of nitrated proteins in TIF of 26-week-old WT SOD1 mice: 150 µg of TIF was loaded into the 2D gel and transferred onto a PDVF membrane. The blot was probed with anti-nitrotyrosine polyclonal antibody (A), after total protein SYPRO Ruby blot staining (B). Spot numbers in (A) correspond to proteins in Table 2. (5.69 MB TIF) [file pone.0008130.s004.tif]

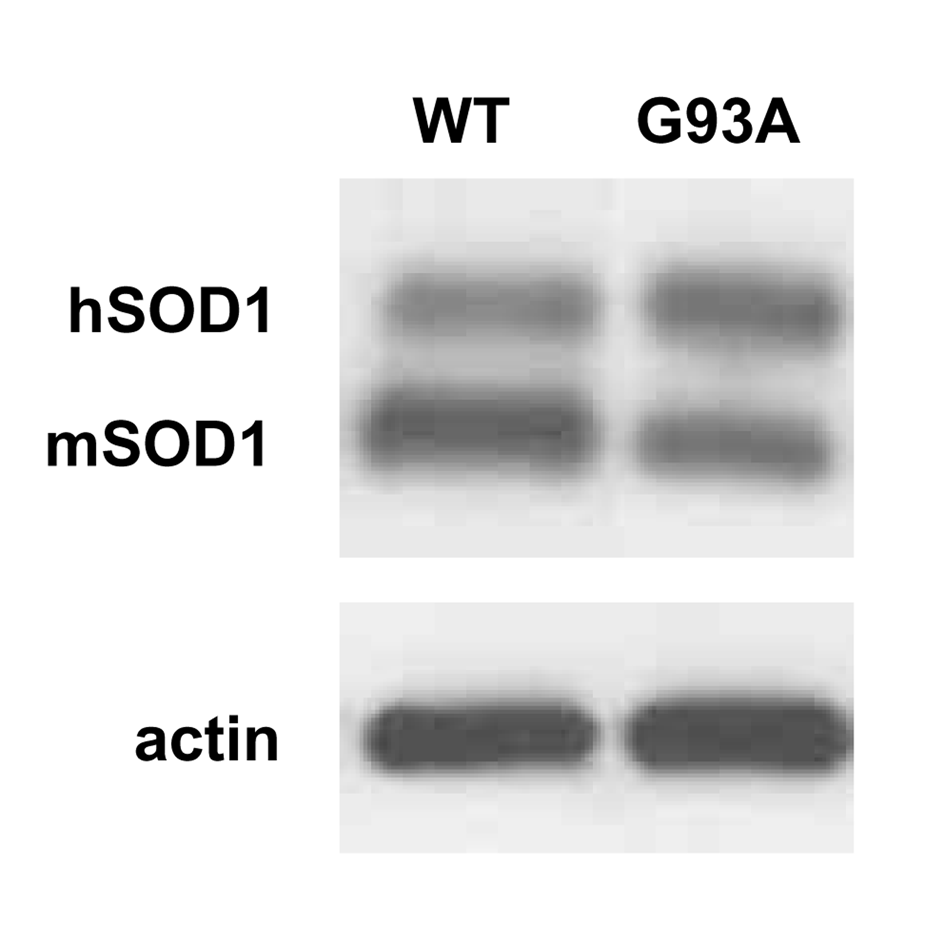

Supplement: Figure S5 — Anti-SOD1 Western blot of total protein extracts from NSC-34 cells expressing WT or G93A SOD1. The same amount of proteins (30 µg) was loaded in immunoblot. (0.90 MB TIF) [file pone.0008130.s005.tif]
