# Supplementary material for: Characterization of Detergent-Insoluble Proteins in ALS Indicates a Causal Link between Nitrative Stress and Aggregation in Pathogenesis
Source: PLoS One. 2009 Dec 2;4(12):e8130. doi: 10.1371/journal.pone.0008130 (PMC2780298; doi:10.1371/journal.pone.0008130)
Supplement: Table S1 — MALDI TOF MS identification of proteins differentially present in TIF from spinal cord of WT and G93A SOD1 mice. (0.12 MB DOC) [file pone.0008130.s006.doc]

#### Table S1. MALDI TOF MS identification of proteins differentially present in TIF from spinal cord of WT and G93A SOD1 mice.

| Spot | Protein name | AC^a^ | Mr_calc_ | pI_calc_ | Mr_obs_ | pI_obs_ | match/  unmatch | cov | | score |
| --- | --- | --- | --- | --- | --- | --- | --- | --- | --- | --- |
| 1 | Neurofilament triplet M protein (NFM)* | P08553 | 95.9 | 4.8 | 220 | 4.4 | 15/42 | 20 | | 77 |
| 2 | Neurofilament triplet H protein (NFH) | P19246 | 116.5 | 5.7 | 200 | 5.3 | 25/34 | 22 | | 131 |
| 3 | Neurofilament triplet L protein (NFL)* | P08551 | 61.4 | 4.6 | 160 | 4.1 | 12/58 | 24 | | 59 |
| 4 | NFM | P08553 | 95.9 | 4.8 | 150 | 4.4 | 26/48 | 27 | | 144 |
| 5 | Glial fibrillary acidic protein (GFAP)* | P03995 | 49.9 | 5.4 | 170 | 5.2 | 16/75 | 34 | | 76 |
| 6 | Endoplasmin | P08113 | 92.4 | 4.7 | 100 | 4.4 | 19/43 | 22 | | 81 |
| 7 | Transitional endoplasmic reticulum ATPase | Q01853 | 89.1 | 5.1 | 100 | 5.1 | 35/38 | 28 | | 167 |
| 8 | Heat shock protein HSP 90-alpha (HSP90) | P07901 | 84.7 | 4.9 | 96.0 | 4.6 | 21/58 | 27 | | 70 |
| 9 | NADH-ubiquinone oxidoreductase 75 kDa subunit | Q91VD9 | 79.7 | 5.5 | 76.2 | 5.2 | 17/51 | 27 | | 82 |
| 10 | Glycerol-3-phosphate dehydrogenase | Q64521 | 80.8 | 6.2 | 78.3 | 6.7 | 17/42 | 22 | | 86 |
| 11 | Aconitase | Q99KI0 | 85.4 | 8.1 | 82.8 | 7.6 | 20/46 | 27 | | 107 |
| 12-13 | Aconitase | Q99KI0 | 85.4 | 8.1 | 83.1 | 7.8 | 14/50 | 18 | | 62 |
| 14 | Aconitase | Q99KI0 | 85.4 | 8.1 | 83.1 | 8.0 | 17/69 | 26 | | 68 |
| 15 | Vimentin* | P20152 | 53.5 | 5.1 | 74.9 | 4.2 | 12/60 | 33 | | 63 |
| 16 | NFL | P08551 | 61.4 | 4.6 | 66.4 | 4.4 | 45/42 | 51 | | 327 |
| 17 | Heat shock cognate 71 kDa protein (HSC70) | P63017 | 70.8 | 5.4 | 67.5 | 5.2 | 26/58 | 40 | | 135 |
| 18 | Protein disulfide-isomerase (PDI) | P09103 | 56.6 | 6.0 | 57.5 | 4.4 | 25/70 | 36 | | 155 |
| 19 | Vimentin* | P20152 | 53.5 | 5.1 | 58.2 | 4.8 | 22/62 | 49 | | 157 |
| 20 | Vimentin* | P20152 | 53.5 | 5.1 | 59.7 | 5.0 | 44/53 | 68 | | 317 |
| 21 | Alpha-internexin | P46660 | 55.8 | 5.2 | 54.7 | 5.2 | 37/31 | 59 | | 351 |
| 22 | Dihydropyrimidinase-related protein 2 | O08553 | 62.1 | 6.0 | 59.1 | 5.7 | 27/58 | 56 | | 183 |
| 23 | Dihydropyrimidinase-related protein 2 | O08553 | 62.1 | 6.0 | 59.3 | 6.1 | 21/60 | 48 | | 130 |
| 24 | Dihydropyrimidinase-related protein 2 | O08553 | 62.1 | 6.0 | 59.7 | 6.4 | 18/61 | 39 | | 104 |
| 25 | Pyruvate kinase M2 | P52480 | 57.7 | 8.4 | 58.0 | 7.7 | 11/32 | 29 | | 73 |
| 26 | Pyruvate kinase M2 | P52480 | 57.7 | 9.4 | 58.0 | 8.1 | 14/69 | 33 | | 71 |
| 27 | Glutamate dehydrogenase 1 | P26443 | 61.3 | 8.1 | 54.8 | 7.6 | 23/43 | 34 | | 112 |
| 28 | Glutamate dehydrogenase 1 | P26443 | 61.3 | 8.1 | 54.8 | 7.9 | 22/59 | 38 | | 110 |
| 29 | ATP synthase alpha chain (ATPase) | Q03265 | 59.7 | 9.2 | 52.5 | 7.1 | 14/69 | 33 | | 71 |
| 30 | ATPase | Q03265 | 59.7 | 9.2 | 52.5 | 7.7 | 18/49 | 43 | | 125 |
| 31 | Vimentin | P20152 | 53.5 | 5.1 | 50 | 4.7 | 26/45 | 55 | | 210 |
| 32 | Alpha-enolase | P17182 | 47.0 | 6.4 | 45.9 | 6.5 | 22/61 | 60 | | 139 |
| 33 | Alpha-enolase | P17182 | 47.0 | 6.4 | 45.0 | 7.0 | 23/61 | 62 | | 161 |
| 34 | Creatine kinase | P30275 | 47.0 | 8.4 | 44.4 | 7.8 | 15/48 | 30 | | 86 |
| 35 | Creatine kinase | P30275 | 47.0 | 8.4 | 45.3 | 8.2 | 12/50 | 28 | | 64 |
| 36 | Vimentin # | P20152 | 53.5 | 5.1 | 40.5 | 4.4 | 15/71 | 38 | | 76 |
| 37 | GFAP # | P03995 | 49.9 | 5.4 | 39.0 | 4.6 | 34/66 | 61 | | 199 |
| 38 | Glutamine synthetase | P15105 | 42.0 | 6.5 | 42.0 | 7.2 | 15/56 | 34 | | 89 |
| 39 | Glutamine synthetase | P15106 | 42.0 | 6.5 | 42.2 | 7.6 | 14/53 | 38 | | 81 |
| 40 | Aspartate aminotransferase | P05201 | 46.1 | 6.8 | 41.5 | 8.2 | 12/71 | 30 | | 63 |
| 41 | GFAP # | P03995 | 49.9 | 5.4 | 38.6 | 4.6 | 30/66 | 58 | | 198 |
| 42 | GFAP # | P03995 | 49.9 | 5.4 | 36.2 | 4.6 | 17/49 | 40 | | 110 |
| 43 | GFAP # | P03995 | 49.9 | 5.4 | 35.3 | 5.4 | 22/65 | 53 | | 145 |
| 44 | Isocitrate dehydrogenase [NAD] subunit alpha | Q9D6R2 | 39.6 | 6.3 | 36.3 | 5.9 | 15/53 | 38 | | 83 |
| 45 | Mitogen-activated protein kinase 1 (ERK2) | P63085 | 41.1 | 6.5 | 37.7 | 7.4 | 10/71 | 34 | | 60 |
| 46 | Fructose-bisphosphate aldolase C (aldolase) | P05063 | 39.2 | 6.8 | 37.8 | 8.1 | 16/42 | 44 | | 131 |
| 47 | Glyceraldehyde-3-phosphate dehydrogenase (GAPDH) | P16858 | 35.7 | 8.5 | 35.4 | 7.4 | 8/48 | 30 | | 53 |
| 48 | GAPDH | P16858 | 35.7 | 8.5 | 35.4 | 7.9 | 10/82 | 38 | | 55 |
| 49 | GAPDH | P16858 | 35.7 | 8.5 | 35.7 | 8.6 | 8/33 |  | 30 | 63 |
| 50 | Pyruvate dehydrogenase E1+ | Q9D051 | 38.9 | 6.4 | 33.3 | 5.4 | 17/44 |  | 43 | 91 |
|  | Guanine nucleotide-binding protein G(I)/G(S)/G(T) subunit beta-2 | P62880 | 37.3 | 5.6 | 33.3 | 5.4 | 15/44 |  | 44 | 86 |
| 51 | L-lactate dehydrogenase B chain (LDH) | P16125 | 36.4 | 5.8 | 33.3 | 5.7 | 19/62 |  | 50 | 118 |
| 52 | Cytosolic malate dehydrogenase | P14152 | 36.4 | 6.2 | 33.3 | 5.4 | 11/62 |  | 35 | 57 |
| 53 | Annexin A5 | P48036 | 35.7 | 4.8 | 28.7 | 4.5 | 9/47 |  | 25 | 60 |
| 54 | 14-3-3 protein gamma | P61982 | 28.2 | 4.8 | 25.8 | 4.4 | 16/78 |  | 43 | 71 |
| 55 | GFAP# | P03995 | 49.9 | 5.4 | 24.9 | 5.2 | 12/37 |  | 27 | 71 |
| 56 | GFAP# | P03995 | 49.9 | 5.4 | 23.4 | 4.8 | 15/65 |  | 38 | 74 |
| 57 | Heat-shock protein beta-1 (HSP27) | P14602 | 21.9 | 6.5 | 23.2 | 6.5 | 10/25 |  | 43 | 109 |
| 58 | NFM# | P08553 | 95.9 | 4.8 | 18.7 | 4.5 | 15/56 |  | 16 | 57 |
| 59 | Alpha crystallin B chain | P23927 | 20.1 | 6.8 | 20.5 | 7.7 | 12/70 |  | 58 | 81 |
| 60 | Alpha crystallin B chain | P23927 | 20.1 | 6.8 | 20.7 | 8.1 | 13/73 |  | 59 | 94 |
| 61 | Peptidyl-prolyl cis-trans isomerase A (CypA) | P17742 | 17.8 | 7.9 | 15.2 | 7.6 | 10/82 |  | 53 | 64 |
| 62 | Superoxide dismutase [Cu-Zn] (SOD1)‡ | P00442 | 15.8 | 5.7 | 17.2 | 5.1 | - |  | - | - |
| 63 | SOD1‡ | P00442 | 15.8 | 5.7 | 17.2 | 5.3 | - |  | - | - |
| 64 | SOD1‡ | P00441 | 15.8 | 5.7 | 17.0 | 5.6 | - |  | - | - |
| 65 | SOD1‡ | P00442 | 15.8 | 5.7 | 17.2 | 5.9 | - |  | - | - |
| 66 | SOD1‡ | P00442 | 15.8 | 5.7 | 17.2 | 6.2 | - |  | - | - |
| a | Laminin subunit beta-2 | Q61292 | 196.2 | 6.3 | 200 | 6.6 | 33/61 |  | 22 | 83 |
| b | Voltage-dependent anion-selective channel protein 1 (VDAC1) | Q60932 | 32.3 | 8.6 | 30.0 | 8.0 | 13/68 |  | 56 | 87 |
| c | GFAP | P03995 | 49.9 | 5.4 | 50.0 | 5.1 | 20/68 |  | 40 | 170 |

^a^AC, accession numbers from SwissProt; Mr_calc_ and pI_calc_, calculated Mr and pI; Mr_obs_ and pI_obs_, observed Mr and pI; score, probability score in Mascot program (database Swissprot, release 57.3, June 2009); cov, percentage of sequence coverage; match/unmatch, matched peptides/unmatched peptides (unmatched peptides include also trypsin autolysis and keratin contaminant fragments); *, modified protein; #, fragment; ‡, identified by 2D WB as previously reported (Basso et al., 2006); a, b, c, proteins not specifically enriched in G93A TIF, but detected as oxidized.
