# Supplementary material for: Characterization of Detergent-Insoluble Proteins in ALS Indicates a Causal Link between Nitrative Stress and Aggregation in Pathogenesis
Source: PLoS One. 2009 Dec 2;4(12):e8130. doi: 10.1371/journal.pone.0008130 (PMC2780298; doi:10.1371/journal.pone.0008130)
Supplement: Table S2 — Proteins enriched in TIF of spinal cord from WT SOD1 mice. (0.04 MB DOC) [file pone.0008130.s007.doc]

Table S2. Proteins enriched in TIF of spinal cord from WT SOD1 mice.

| Spot | Protein name | AC^a^ | WT^b^ | G93A^c^ | FC^d^ |
| --- | --- | --- | --- | --- | --- |
| 1 | NFM* | P08553 | 11.6±1.9 | 6.4±0.9 | 1.8 |
| 2 | NFH | P19246 | 46.3±6.3 | 21.8±4.4 | 2.1 |
| 3 | NFL* | P08551 | 38.3±4.04 | 17.7±4.8 | 2.1 |
| 4 | NFM | P08553 | 69.0±18.9 | 43.2±3.6 | 1.6 |
| 15 | Vimentin* | P20152 | 4.1±1.9 | 1.3±0.3 | 3.1 |
| 16 | NFL | P08551 | 138.6±19.5 | 64.3±12.1 | 2.1 |
| 19 | Vimentin* | P20152 | 5.7±1.3 | 2.6±0.7 | 2.2 |
| 21 | Alpha-internexin | P46660 | 69.3±10.9 | 36.6±8.7 | 1.9 |

AC^a^, accession numbers from SwissProt; ^b^WT, normalised spot volumes of the WT sample, the mean of three replicates ± SD; ^c^G93A, normalised spot volumes of the G93A sample, the mean of three replicates ± SD; ^d^FC, fold change of spot volume as ratio of the spot volumes (WT/G93A); *, modified protein.
