# Supplementary material for: Characterization of Detergent-Insoluble Proteins in ALS Indicates a Causal Link between Nitrative Stress and Aggregation in Pathogenesis
Source: PLoS One. 2009 Dec 2;4(12):e8130. doi: 10.1371/journal.pone.0008130 (PMC2780298; doi:10.1371/journal.pone.0008130)
Supplement: Table S3 — WB of selected proteins. The proteins were measured in TIF (aggregate) and in the soluble (soluble) fraction of spinal cord protein extracts from WT and G93A SOD1 mice at 26 weeks of age. (0.11 MB DOC) [file pone.0008130.s008.doc]

Table S3. WB of selected proteins. The proteins were measured in TIF (aggregate) and in the soluble (soluble) fraction of spinal cord protein extracts from WT and G93A SOD1 mice at 26 weeks of age.

| **Protein name** | **WT^a^** | **G93A^b^** | **Fold^c^** | **WT^a^** | **G93A^b^** | **FC^c^** |
| --- | --- | --- | --- | --- | --- | --- |
|  | **Aggregate** | | | **Soluble** | | |
| HSP90 |  | + |  | 1.4±0.2 | 1.8±1.2 | 1.3 |
| Aconitase | 0.1±0.0 | 2.0±1.1 | 20.0* | 1.0±0.1 | 1.0±0.1 | 1.0 |
| HSC70 | 0.5±0.1 | 1.4±0.4 | 2.8* | 1.5±0.3 | 0.5±0.2 | 0.3* |
| NFL | 4.1±0.1 | 1.9±0.6 | 0.5* | 0.1±0.0 | 0.05±0.0 | 0.5* |
| ERK1/2 | 0.3±0.1 | 1.7±0.8 | 5.7* | 1.0±0.2 | 1.1±0.3 | 1.1 |
| MAPKp38 | 0.8±0.1 | 1.1±0.1 | 1.4* | 0.9±0.1 | 1.1±0.5 | 1.2 |
| 14-3-3 protein gamma |  | + |  | 1.2±0.3 | 1.0±0.5 | 0.8 |
| CypA |  | + |  | 1.4±0.2 | 0.7±0.1 | 0.5* |

^a^WT, normalized immunoreactivity of the WT samples, mean ± SD, n=3; ^b^G93A, normalized immunoreactivity of the G93A samples, mean ± SD, n=3; ^*^, *p* < 0.05 by Student’s *t*-test; ^c^FC, fold change of immunoreactivity as ratio of the normalized immunoreactivity (G93A/WT); +, detected only in G93A samples.

**Table S4.** DIGE analysis of TIF from spinal cord of WT and G93A SOD1 mice at different disease stages.

| **Spot** | **Protein name** | **WT,26^a^** | **G93A,12^b^** | | **G93A,17^c^** | | **G93A,26^d^** | **Age of aggregation^e^** | |
| --- | --- | --- | --- | --- | --- | --- | --- | --- | --- |
|  | **Cytoskeleton** | | | | | | | | |
| 1 | NFM* | 1,7 | 1,8 | 1,3 | | 0,3 | | |  |
| 2 | NFH | 0,5 | 1,9 | 1,3 | | 0,4 | | | 12,17 |
| 3 | NFL* | 0,7 | 1,0 | 0,4 | | 0,3 | | | 12 |
| 4 | NFM | 0,4 | 0,9 | 1,5 | | 0,2 | | | 12,17 |
| 5 | GFAP* | 0,9 | 1,0 | 0,9 | | 1,7 | | | 26 |
| 15 | Vimentin* | 0,5 | 1,1 | 1,6 | | 0,3 | | | 12,17 |
| 16 | NFL | 0,5 | 0,7 | 1,3 | | 0,2 | | | 12,17 |
| 19 | Vimentin* | 7,3 | 1,2 | 1,1 | | 0,9 | | |  |
| 20 | Vimentin* | 0,0 | 1,1 | 1,1 | | 1,2 | | | 12,17,26 |
| 21 | Alpha-internexin | 0,6 | 1,4 | 1,3 | | 0,3 | | | 12,17 |
| 31 | Vimentin | 1,0 | 0,7 | 0,8 | | 0,8 | | |  |
| 36 | Vimentin^#^ | - | 1,4 | 2,2 | | 1,2 | | | 12,17,26 |
| 37 | GFAP ^#^ | 1,1 | 1,0 | 1,0 | | 0,4 | | | 12,17,26 |
| 41 | GFAP ^#^ | 1,1 | 1,0 | 1,0 | | 0,7 | | |  |
| 42 | GFAP ^#^ | 0,7 | 1,2 | 1,5 | | 0,7 | | | 12,17 |
| 43 | GFAP ^#^ | - | 0,6 | 0,7 | | 2,8 | | | 12,17,26 |
| 55 | GFAP ^#^ | - | 1,2 | 1,8 | | 3,5 | | | 12,17,26 |
| 56 | GFAP ^#^ | - | 0,2 | 0,1 | | 3,3 | | | 12,17,26 |
| 58 | NFM^#^ | - | 0,9 | 1,2 | | 1,3 | | | 12,17,26 |
|  | **Metabolism** | | | | | | | | |
| 25 | Pyruvate kinase M2 | 0,7 | 0,4 | 0,9 | | 3,3 | | | 26 |
| 26 | Pyruvate kinase M2 | 0,6 | 0,7 | 1,2 | | 2,2 | | | 17,26 |
| 32 | Alpha enolase | - | 0,8 | 0,7 | | 2,8 | | | 12,17,26 |
| 33 | Alpha enolase | - | 0,8 | 0,9 | | 3,6 | | | 12,17,26 |
| 38 | Glutamine synthetase | 1,1 | 0,3 | 0,4 | | 6,9 | | | 26 |
| 39 | Glutamine synthetase | 1,6 | 0,6 | 0,5 | | 3,8 | | | 26 |
| 40 | Aspartate aminotransferase | 1,3 | 1,0 | 1,2 | | 2,7 | | | 26 |
| 46 | Aldolase C | 1,5 | 1,0 | 1,0 | | 3,8 | | | 26 |
| 47 | GAPDH | 0,4 | 0,2 | 0,6 | | 4,0 | | | 17,26 |
| 48 | GAPDH | 1,2 | 0,3 | 0,4 | | 3,5 | | | 26 |
| 49 | GAPDH | 1,5 | 0,5 | 0,5 | | 3,4 | | | 26 |
| 51 | LDH | 0,6 | 0,8 | 1,0 | | 1,8 | | | 12,17,26 |
| 52 | Cytosolic malate dehydrogenase | - | - | 1,4 | | 3,1 | | | 17,26 |
|  | **Mitochondria** | | | | | | | | |
| 9 | NADH-ubiquinone oxidoreductase | 0,6 | 1,6 | 1,1 | | 1,6 | | | 12,17,26 |
| 10 | Glycerol-3-phosphate dehydrogenase | 1,1 | 1,3 | 0,6 | | 2,0 | | | 26 |
| 11 | Aconitase | 0,2 | 0,7 | 0,3 | | 1,9 | | | 12,17,26 |
| 12-13 | Aconitase | 0,4 | 0,3 | 0,4 | | 2,6 | | | 26 |
| 14 | Aconitase | 0,8 | 1,2 | 0,7 | | 3,2 | | | 12,26 |
| 27 | Glutamate dehydrogenase 1 | 0,5 | 0,6 | 0,3 | | 3,0 | | | 26 |
| 28 | Glutamate dehydrogenase 1 | 0,9 | 1,0 | 0,4 | | 2,7 | | | 26 |
| 29 | ATPase | 0,4 | 0,2 | 0,4 | | 3,9 | | | 26 |
| 30 | ATPase | 0,3 | 0,3 | 0,3 | | 2,9 | | | 26 |
| 34 | Creatine kinase | 0,4 | 0,4 | 0,5 | | 4,7 | | | 26 |
| 35 | Creatine kinase | 0,9 | 0,8 | 0,6 | | 4,7 | | | 26 |
| 44 | Isocitrate DH [NAD] subunit alpha | - | - | 1,0 | | 3,3 | | | 17,26 |
| 50 | Pyruvate dehydrogenase E1 | 0,5 | 0,8 | 0,5 | | 1,8 | | | 12,26 |
|  | **Chaperones** | | | | | | | | |
| 8 | HSP90 | - | 1,3 | 4,5 | | 2,4 | | | 12,17,26 |
| 17 | HSC70 | 0,7 | 1,5 | 1,1 | | 1,0 | | | 12,17,26 |
| 57 | HSP27 | - | - | 1,6 | | 0,9 | | | 17,26 |
| 59 | Alpha crystallin B chain | - | 1,5 | 1,8 | | 2,7 | | | 12,17,26 |
| 60 | Alpha crystallin B chain | - | 0,7 | 1,1 | | 2,2 | | | 12,17,26 |
| 61 | CypA | - | - | - | | 0,4 | | | 26 |
|  | **Signaling** | | | | | | | | |
| 45 | ERK2 | 1,6 | 0,9 | 0,6 | | 3,0 | | | 26 |
| 53 | Annexin A5 | 0,9 | 2,6 | 1,5 | | 3,4 | | | 12,17,26 |
| 54 | 14-3-3 protein gamma | - | 0,9 | 1,1 | | 2,9 | | | 12,17,26 |
|  | **Endoplasmic reticulum** | | | | | | | | |
| 6 | Endoplasmin | - | 1,9 | 2,1 | | 0,3 | | | 12,17,26 |
| 7 | Transitional ER ATPase | - | 1,5 | 1,3 | | 1,0 | | | 12,17,26 |
| 18 | PDI | 1,5 | 1,6 | 1,1 | | 2,0 | | | 26 |
|  | **Others** | | | | | | | | |
| 22 | Dihydropyrimidinase-related protein 2 | 0,4 | 0,7 | 0,4 | | 0,8 | | | 12,26 |
| 23 | Dihydropyrimidinase-related protein 2 | 0,7 | 1,3 | 0,4 | | 1,5 | | | 12,26 |
| 24 | Dihydropyrimidinase-related protein 2 | 0,7 | 1,0 | 0,5 | | 1,0 | | | 12,26 |
| 62 | SOD1 | - | - | 2,2 | | 3,2 | | | 17,26 |
| 63 | SOD1 | - | 0,1 | 0,5 | | 5,8 | | | 12,17,26 |
| 64 | SOD1 | - | 0,1 | 1,9 | | 3,1 | | | 12,17,26 |
| 65 | SOD1 | - | 0,4 | 1,5 | | 1,6 | | | 12,17,26 |
| 66 | SOD1 | - | 0,2 | 0,4 | | 3,9 | | | 12,17,26 |

^a^WT, normalized spot volumes of the WT sample ; ^b,c,d^G93A, normalized spot volumes of the G93A samples from mice at 12, 17 and 26 weeks of age. Values are mean of two DIGE experiments with dye-swapping. ^e^age of aggregation, age of the mice at which the protein is considered aggregated: normalized spot volume ratio G93A/WT >1.4 fold; -, spot not detected; *, Mr higher than expected, unknown protein modification; ^#^, Mr lower than expected, possible protein fragment.
