# Supplementary material for: Characterization of Detergent-Insoluble Proteins in ALS Indicates a Causal Link between Nitrative Stress and Aggregation in Pathogenesis
Source: PLoS One. 2009 Dec 2;4(12):e8130. doi: 10.1371/journal.pone.0008130 (PMC2780298; doi:10.1371/journal.pone.0008130)
Supplement: Table S4 — DIGE analysis of TIF from spinal cord of WT and G93A SOD1 mice at different disease stages. (0.10 MB DOC) [file pone.0008130.s009.doc]

**Table S4.** DIGE analysis of TIF from spinal cord of WT and G93A SOD1 mice at different disease stages.

| **Spot** | **Protein name** | **WT,26^a^** | **G93A,12^b^** | | **G93A,17^c^** | | **G93A,26^d^** | **Age of aggregation^e^** | |
| --- | --- | --- | --- | --- | --- | --- | --- | --- | --- |
|  | **Cytoskeleton** | | | | | | | | |
| 1 | NFM* | 1,7 | 1,8 | 1,3 | | 0,3 | | |  |
| 2 | NFH | 0,5 | 1,9 | 1,3 | | 0,4 | | | 12,17 |
| 3 | NFL* | 0,7 | 1,0 | 0,4 | | 0,3 | | | 12 |
| 4 | NFM | 0,4 | 0,9 | 1,5 | | 0,2 | | | 12,17 |
| 5 | GFAP* | 0,9 | 1,0 | 0,9 | | 1,7 | | | 26 |
| 15 | Vimentin* | 0,5 | 1,1 | 1,6 | | 0,3 | | | 12,17 |
| 16 | NFL | 0,5 | 0,7 | 1,3 | | 0,2 | | | 12,17 |
| 19 | Vimentin* | 7,3 | 1,2 | 1,1 | | 0,9 | | |  |
| 20 | Vimentin* | 0,0 | 1,1 | 1,1 | | 1,2 | | | 12,17,26 |
| 21 | Alpha-internexin | 0,6 | 1,4 | 1,3 | | 0,3 | | | 12,17 |
| 31 | Vimentin | 1,0 | 0,7 | 0,8 | | 0,8 | | |  |
| 36 | Vimentin^#^ | - | 1,4 | 2,2 | | 1,2 | | | 12,17,26 |
| 37 | GFAP ^#^ | 1,1 | 1,0 | 1,0 | | 0,4 | | | 12,17,26 |
| 41 | GFAP ^#^ | 1,1 | 1,0 | 1,0 | | 0,7 | | |  |
| 42 | GFAP ^#^ | 0,7 | 1,2 | 1,5 | | 0,7 | | | 12,17 |
| 43 | GFAP ^#^ | - | 0,6 | 0,7 | | 2,8 | | | 12,17,26 |
| 55 | GFAP ^#^ | - | 1,2 | 1,8 | | 3,5 | | | 12,17,26 |
| 56 | GFAP ^#^ | - | 0,2 | 0,1 | | 3,3 | | | 12,17,26 |
| 58 | NFM^#^ | - | 0,9 | 1,2 | | 1,3 | | | 12,17,26 |
|  | **Metabolism** | | | | | | | | |
| 25 | Pyruvate kinase M2 | 0,7 | 0,4 | 0,9 | | 3,3 | | | 26 |
| 26 | Pyruvate kinase M2 | 0,6 | 0,7 | 1,2 | | 2,2 | | | 17,26 |
| 32 | Alpha enolase | - | 0,8 | 0,7 | | 2,8 | | | 12,17,26 |
| 33 | Alpha enolase | - | 0,8 | 0,9 | | 3,6 | | | 12,17,26 |
| 38 | Glutamine synthetase | 1,1 | 0,3 | 0,4 | | 6,9 | | | 26 |
| 39 | Glutamine synthetase | 1,6 | 0,6 | 0,5 | | 3,8 | | | 26 |
| 40 | Aspartate aminotransferase | 1,3 | 1,0 | 1,2 | | 2,7 | | | 26 |
| 46 | Aldolase C | 1,5 | 1,0 | 1,0 | | 3,8 | | | 26 |
| 47 | GAPDH | 0,4 | 0,2 | 0,6 | | 4,0 | | | 17,26 |
| 48 | GAPDH | 1,2 | 0,3 | 0,4 | | 3,5 | | | 26 |
| 49 | GAPDH | 1,5 | 0,5 | 0,5 | | 3,4 | | | 26 |
| 51 | LDH | 0,6 | 0,8 | 1,0 | | 1,8 | | | 12,17,26 |
| 52 | Cytosolic malate dehydrogenase | - | - | 1,4 | | 3,1 | | | 17,26 |
|  | **Mitochondria** | | | | | | | | |
| 9 | NADH-ubiquinone oxidoreductase | 0,6 | 1,6 | 1,1 | | 1,6 | | | 12,17,26 |
| 10 | Glycerol-3-phosphate dehydrogenase | 1,1 | 1,3 | 0,6 | | 2,0 | | | 26 |
| 11 | Aconitase | 0,2 | 0,7 | 0,3 | | 1,9 | | | 12,17,26 |
| 12-13 | Aconitase | 0,4 | 0,3 | 0,4 | | 2,6 | | | 26 |
| 14 | Aconitase | 0,8 | 1,2 | 0,7 | | 3,2 | | | 12,26 |
| 27 | Glutamate dehydrogenase 1 | 0,5 | 0,6 | 0,3 | | 3,0 | | | 26 |
| 28 | Glutamate dehydrogenase 1 | 0,9 | 1,0 | 0,4 | | 2,7 | | | 26 |
| 29 | ATPase | 0,4 | 0,2 | 0,4 | | 3,9 | | | 26 |
| 30 | ATPase | 0,3 | 0,3 | 0,3 | | 2,9 | | | 26 |
| 34 | Creatine kinase | 0,4 | 0,4 | 0,5 | | 4,7 | | | 26 |
| 35 | Creatine kinase | 0,9 | 0,8 | 0,6 | | 4,7 | | | 26 |
| 44 | Isocitrate DH [NAD] subunit alpha | - | - | 1,0 | | 3,3 | | | 17,26 |
| 50 | Pyruvate dehydrogenase E1 | 0,5 | 0,8 | 0,5 | | 1,8 | | | 12,26 |
|  | **Chaperones** | | | | | | | | |
| 8 | HSP90 | - | 1,3 | 4,5 | | 2,4 | | | 12,17,26 |
| 17 | HSC70 | 0,7 | 1,5 | 1,1 | | 1,0 | | | 12,17,26 |
| 57 | HSP27 | - | - | 1,6 | | 0,9 | | | 17,26 |
| 59 | Alpha crystallin B chain | - | 1,5 | 1,8 | | 2,7 | | | 12,17,26 |
| 60 | Alpha crystallin B chain | - | 0,7 | 1,1 | | 2,2 | | | 12,17,26 |
| 61 | CypA | - | - | - | | 0,4 | | | 26 |
|  | **Signaling** | | | | | | | | |
| 45 | ERK2 | 1,6 | 0,9 | 0,6 | | 3,0 | | | 26 |
| 53 | Annexin A5 | 0,9 | 2,6 | 1,5 | | 3,4 | | | 12,17,26 |
| 54 | 14-3-3 protein gamma | - | 0,9 | 1,1 | | 2,9 | | | 12,17,26 |
|  | **Endoplasmic reticulum** | | | | | | | | |
| 6 | Endoplasmin | - | 1,9 | 2,1 | | 0,3 | | | 12,17,26 |
| 7 | Transitional ER ATPase | - | 1,5 | 1,3 | | 1,0 | | | 12,17,26 |
| 18 | PDI | 1,5 | 1,6 | 1,1 | | 2,0 | | | 26 |
|  | **Others** | | | | | | | | |
| 22 | Dihydropyrimidinase-related protein 2 | 0,4 | 0,7 | 0,4 | | 0,8 | | | 12,26 |
| 23 | Dihydropyrimidinase-related protein 2 | 0,7 | 1,3 | 0,4 | | 1,5 | | | 12,26 |
| 24 | Dihydropyrimidinase-related protein 2 | 0,7 | 1,0 | 0,5 | | 1,0 | | | 12,26 |
| 62 | SOD1 | - | - | 2,2 | | 3,2 | | | 17,26 |
| 63 | SOD1 | - | 0,1 | 0,5 | | 5,8 | | | 12,17,26 |
| 64 | SOD1 | - | 0,1 | 1,9 | | 3,1 | | | 12,17,26 |
| 65 | SOD1 | - | 0,4 | 1,5 | | 1,6 | | | 12,17,26 |
| 66 | SOD1 | - | 0,2 | 0,4 | | 3,9 | | | 12,17,26 |

^a^WT, normalized spot volumes of the WT sample ; ^b,c,d^G93A, normalized spot volumes of the G93A samples from mice at 12, 17 and 26 weeks of age. Values are mean of two DIGE experiments with dye-swapping. ^e^age of aggregation, age of the mice at which the protein is considered aggregated: normalized spot volume ratio G93A/WT >1.4 fold; -, spot not detected; *, Mr higher than expected, unknown protein modification; ^#^, Mr lower than expected, possible protein fragment.
