# Supplementary material for: Characterization of Detergent-Insoluble Proteins in ALS Indicates a Causal Link between Nitrative Stress and Aggregation in Pathogenesis
Source: PLoS One. 2009 Dec 2;4(12):e8130. doi: 10.1371/journal.pone.0008130 (PMC2780298; doi:10.1371/journal.pone.0008130)
Supplement: Table S5 — Clinical and neuropathological characteristics of ALS cases. (0.03 MB DOC) [file pone.0008130.s010.doc]

**Table S5.** Clinical and neuropathological characteristics of ALS cases.

| 1* | 39 y/o male, rapidly progressive course, death from respiratory failure, pathology demonstrate neurofilament (NF) aggregate formation in motor neurons, degeneration of motor neurons in spinal cord and brainstem, with corticospinal tract (CST) degeneration; ubiquitinated inclusions present. |
| --- | --- |
| 2* | 78 y/o male. 1 year duration, clinically and neuropathologically ALS with inclusion formation (NF, ubiquitin). |
| 3* | 55 y/o male, slowly progressive variant with initial lower motor neuron predominant features, ultimately upper motor neuron present, duration approx. 10 years; loss of CST and motor neurons throughout brainstem and spinal cord; considerable gliosis given the disease duration; few ubiquitin positive inclusions; markedly atrophic ventral roots. |
| 4* | 72 y/o male, approx. 3 year history, frontal and temporal cortex show mild superficial linear spongiosis, loss of Betz cells, pallor of CST, prominent ubiquitin inclusions in motor neurons both brainstem and spinal cord. |
| 5* | 54 y/o male, 3 year duration with behavioural presentation; severe loss of motor neurons, with remaining showing ubiquitin inclusions and eosinophilic bodies (Bunina), prominent loss of CST. |
| 6 | 67 y/o male, 3 years duration with upper and lower motor neuron involvement at  presentation. Ubiquitin- and TDP-43-positive cytoplasmic inclusions in motor neurons throughout spinal cord and brainstem. |
| 7 | 55 y/o male, clinically ALS and behavioral symptoms. 4 years history. Loss of motor neurons in spinal cord; ubiquitinated and TDP-43-positive inclusions in motor neurons. |

All cases were negative for mutations in TDP-43 and SOD1. *1-5 cases were tested also for mutations in FUS/TLS and were negative. For 1-5 cases TDP-43 immunohistochemistry data are not available.
